# Multi-organ impairment and long COVID: a 1-year prospective, longitudinal cohort study

**DOI:** 10.1177/01410768231154703

**Published:** 2023-02-14

**Authors:** Andrea Dennis, Daniel J Cuthbertson, Dan Wootton, Michael Crooks, Mark Gabbay, Nicole Eichert, Sofia Mouchti, Michele Pansini, Adriana Roca-Fernandez, Helena Thomaides-Brears, Matt Kelly, Matthew Robson, Lyth Hishmeh, Emily Attree, Melissa Heightman, Rajarshi Banerjee, Amitava Banerjee

**Affiliations:** 1 Perspectum, Oxford, OX4 2LL, UK; 2 Institute of Cardiovascular and Metabolic Medicine, University of Liverpool, Liverpool, L7 8TX, UK; 3 Institute of Infection, Veterinary and Ecological Sciences, University of Liverpool, Liverpool, CH64 7TE, UK; 4 Department of Respiratory Medicine, Hull and East Yorkshire Hospitals NHS Foundation Trust, Hull, HU3 2JZ, UK; 5 Institute of Clinical and Applied Health Research, University of Hull, Hull, HU6 7RX, UK; 6 Institute of Population Health, University of Liverpool, Waterhouse Building, Block B, Brownlow Street, Liverpool, L69 3GF, UK; 7 Department of Radiology, John Radcliffe Hospital, Oxford University Hospitals NHS Foundation Trust, OX3 0AG, Oxford, UK; 8 Long Covid SOS, Faringdon, Oxfordshire, SN7 7EX, UK; 9 UKDoctors#Longcovid, London, N1 5HZ, UK; 10 Department of Medicine, University College London Hospitals NHS Foundation Trust, London, NW1 2BU, UK; 11 Institute of Health Informatics, University College London, London, NW1 2DA, UK; 12 Department of Cardiology, Barts Health NHS Trust, London, EC1A 7BE, UK

**Keywords:** COVID-19, long COVID, organ impairment, quality of life, prevention, integrated care

## Abstract

**Objectives:**

To determine the prevalence of organ impairment in long COVID patients at 6 and 12 months after initial symptoms and to explore links to clinical presentation.

**Design:**

Prospective cohort study.

**Participants:**

Individuals.

**Methods:**

In individuals recovered from acute COVID-19, we assessed symptoms, health status, and multi-organ tissue characterisation and function.

**Setting:**

Two non-acute healthcare settings (Oxford and London). Physiological and biochemical investigations were performed at baseline on all individuals, and those with organ impairment were reassessed.

**Main outcome measures:**

Primary outcome was prevalence of single- and multi-organ impairment at 6 and 12 months post COVID-19.

**Results:**

A total of 536 individuals (mean age 45 years, 73% female, 89% white, 32% healthcare workers, 13% acute COVID-19 hospitalisation) completed baseline assessment (median: 6 months post COVID-19); 331 (62%) with organ impairment or incidental findings had follow-up, with reduced symptom burden from baseline (median number of symptoms 10 and 3, at 6 and 12 months, respectively). Extreme breathlessness (38% and 30%), cognitive dysfunction (48% and 38%) and poor health-related quality of life (EQ-5D-5L < 0.7; 57% and 45%) were common at 6 and 12 months, and associated with female gender, younger age and single-organ impairment. Single- and multi-organ impairment were present in 69% and 23% at baseline, persisting in 59% and 27% at follow-up, respectively.

**Conclusions:**

Organ impairment persisted in 59% of 331 individuals followed up at 1 year post COVID-19, with implications for symptoms, quality of life and longer-term health, signalling the need for prevention and integrated care of long COVID.

Trial Registration: ClinicalTrials.gov Identifier: NCT04369807

## Background

Symptoms of long COVID, also known as post-acute sequelae of COVID-19, are well documented,^1,2^ but natural history is poorly characterised, either by symptoms, organ impairment or function.^3–5^ Among 3762 individuals with suspected or confirmed COVID-19, debilitating symptoms lasted beyond 35 weeks, with fatigue, breathlessness and cognitive dysfunction being the most frequent.^6^ In the UK’s largest long COVID clinic, non-hospitalised patients required specialist referral at similar rates to hospitalised patients and were more likely to report breathlessness and fatigue, with reduced health-related quality of life (HRQoL).^7^ A US study of 270,000 individuals post COVID-19 showed that one-third had persistent symptoms at 3–6 months (more common than post-influenza symptoms based on a matched cohort with otherwise similar risk factors), possibly due to direct organ-specific rather than general viral effects, and potentially informing development of effective treatments.^8^

Long COVID may be linked to severity of initial illness in some hospitalised patients, but prognostic factors are neither defined nor investigated systematically in non-hospitalised patients.^7–9^ To conduct trials of possible therapies for long COVID, we need stratification by symptoms or investigations.^3^ Our interim magnetic resonance imaging (MRI) data in 201 individuals showed mild organ impairment in the heart, lungs, kidneys, liver, pancreas and spleen, with single- and multi-organ impairment in 70% and 29%, respectively, 4 months after COVID-19.^9^ Clinical utility of these MRI metrics for chronic and multi-system conditions has been shown.^10^^,11^ More severe ongoing symptoms of breathlessness and fatigue were associated with myocarditis (*p* < 0.05)^9^, but symptoms and multi-organ manifestations have not been correlated.

In the UK and other countries, health system and research responses have begun at scale.^12^ However, clinical patient pathways are unclear and there are still no proven, evidence-based therapies, either in subgroups or in the overall long COVID population. Single- and multi-organ impairments need investigation over the medium and long term to assess resource utilisation and health system needs.

In individuals with long COVID, we therefore prospectively investigated the following:

1. Symptoms, organ impairment and function over 1 year, particularly relating to ongoing breathlessness, cognitive dysfunction and HRQoL.
2. Associations between symptoms and organ impairment.

## Methods

### Patient population and study design

This study took place during the COVID-19 emergency, before the formal definition of long COVID. Patients with evidence of COVID-19 who no longer had active SARS-CoV-2 infection, but had ongoing symptoms, could enter the study. A retrospective evaluation of the duration of the symptoms defined individuals who had long COVID based on continuing symptoms for ≥12 weeks, as informed by the earliest WHO, NICE and NHS England guidance regarding post-COVID complications that are now in current guidelines, policy and practice in the UK.^7^

Recruitment was by response to advertisement or specialist referral to two non-acute imaging sites (Perspectum, Oxford and Mayo Clinic Healthcare, London) from April 2020 to August 2021 (the period of COVID-19 waves 1 and 2 in UK) and written informed consent was provided (see Supplementary Methods for inclusion and exclusion criteria). Those with evidence of organ impairment, based on bloods, MRI or incidental findings (organ abnormalities such as lesions, cysts, abnormal vessels and tumours incidentally detected during the MRI analysis), were invited for a 6-month follow-up (contacted on ≥2 occasions to minimise loss to follow-up). Each visit comprised MRI and blood investigations (full blood count, biochemistry) and online questionnaires completed beforehand.^9^

## Diagnostic assessment in non-acute settings

Quantitative multi-organ MRI (COVERSCAN, Perspectum, Oxford) was used to assess organ impairment as previously reported (Figure S1),^9^ using healthy controls (no prior COVID-19 diagnosis, no hospital discharge ≥4 months prior to enrolment and contraindications to MRI; 59 in Oxford, 33 in London). Participants underwent a 40-min MRI scan of the lungs, heart, kidney, liver, pancreas and spleen on 1.5-T or 3-T Siemens scanners at three imaging sites (Oxford: MAGNETOM Aera 1.5 T, Mayo Healthcare London: MAGNETOM Vida 3 T, Chenies Mews Imaging Centre London: MAGNETOM Prisma 3 T). MR metrics were standardised to deliver a single report interpretable by clinicians. Each report included 49 organ-specific metrics with reference ranges to determine impairment (updated from our prior study^10^) after determining distribution of each metric in healthy controls matched for age and sex (*n* = 92) and for organ volumes from healthy controls representing complete sex and height subgroups (*N* = 1835) in this study and UK Biobank^13^ (Tables S1a and S1b). Repeatability of the metrics was evaluated in the healthy controls using standardised performance testing criteria. Technical success was determined by reporting quality-assured measures for each variable reported herein, and overall, in delivering a report for each patient.

## Symptoms, function and organ impairment

Assessment focused on commonly reported symptoms, HRQoL^9^ and degree of breathlessness (Dyspnoea 12 scale^7^). Participants were asked at follow-up about time off work due to COVID-19 (not done at baseline). Multi-organ impairment at baseline and follow-up was defined as ≥2 MRI measurements from different organs outside reference ranges. Further details are in Supplementary Methods.

## Statistical analysis

The study was powered based on the primary outcome measure: to determine the prevalence of heart, kidney and liver injury in a cohort of patients recovering from COVID-19, using multi-parametric MRI, defined relative to pre-defined thresholds from a healthy cohort. Using a published method,^14^ the required sample size for 10% expected prevalence,^15^ 5% allowable margin of error and 95% confidence and allowing for 10% attrition rate, was 507. Analyses were conducted in R version 4.1.0, defining statistical significance by uncorrected *p*-value <0.05 (2-sided). Normally distributed-continuous variables are expressed as mean (standard deviation, SD); non-normally distributed-continuous are expressed as median (interquartile range, IQR); and categorical variables are expressed in frequency (percentage). Fisher’s exact or McNemar’s tests were used for unpaired and paired categorical data, and paired/unpaired t-test or Wilcoxon tests were used for paired/unpaired continuous data, depending on the normality of the underlying distribution. Stepwise multi-variable logistic regression was performed for associations with symptom groups, and a sensitivity analysis excluding metabolic syndrome was performed (Supplementary Methods).

## Results

### Study population for diagnostic assessment in non-acute settings

**Figure 1.**
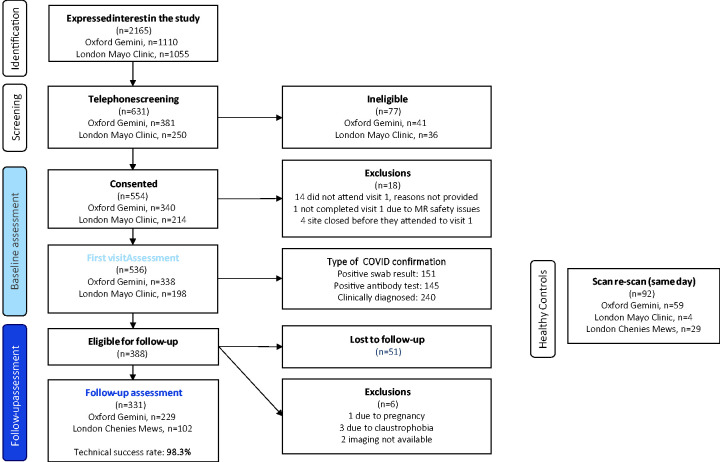
Study population from recruitment to follow-up.

A total of 536 individuals (mean age 45 years, 73% female, median body mass index (BMI) 25 kg/m^2^, 13% COVID-19 hospitalisation, 32% healthcare workers) were included at baseline (Figure 1). Demographics were comparable to healthy controls (Table S1a). Most were ‘UK first wave’ (COVID-19 January–September 2020: *n* = 497) and 39 were ‘second wave’ (COVID-19 after September 2020). Over half of individuals (*n* = 296, 55%) had experienced acute COVID-19 confirmed by antibody or PCR tests and they had marginally higher BMI (25 kg/m^2^, 95% confidence interval (CI) 22–28) compared to those with clinical diagnosis of COVID-19 (26 kg/m^2^, 95% CI 23–30) (Table S2). Median time from initial COVID-19 symptoms to baseline assessment was 182 days (IQR: 132–222).

A total of 388 individuals (72%) with organ impairment identified at baseline, from blood or MRI assessment, were eligible for follow-up, of which 331 (62% of baseline cohort) completed (the follow-up group). Median time from baseline to follow-up assessment was 196 days (IQR: 182–209). Age and BMI were higher in those who completed both visits, and more had been hospitalised for acute COVID-19, compared to those without organ impairment who were not eligible for follow-up (Table S3). In the follow-up group, demographics and risk factors showed no major differences between baseline and follow-up (Table 1). Technical success of MRI and integrated in-person assessment was 99.1% and 98.3% at baseline and follow-up assessments, respectively. Reports summarising findings were delivered to patients and primary care clinicians in all cases.

**Table 1.** Characteristics of individuals with long COVID at baseline and for follow-up.

	Whole cohort	Follow-up group		
Characteristic	Baseline(*n* = 536)	Baseline(*n* = 331)	Follow-up(*n* = 331)	*p* value (baseline vs. follow-up visits)
Age (years)	45 (11)	46 (11)	47 (11)	–
Sex (*n* females)	389 (73%)	241 (73%)	241 (73%)	–
BMI (kg/m^2^)	25 (23, 29)	26 (23, 30)	26 (23,31)	0.269
Ethnicity				–
White	477 (89%)	295 (89%)	295 (89%)	
Mixed	21 (4%)	12 (4%)	3 (1%)	
South Asian	24 (4%)	17 (5%)	15 (5%)	
Black	13 (2%)	6 (2%)	5 (2%)	
Healthcare worker	172 (32%)	112 (34%)	112 (34%)	No change
At least one COVID-19 vaccination	10 (2%)	5 (2%)	197 (60%)	<0.001
Co-morbidities and risks				
Smoking				
Never	349 (65%)	218 (66%)	222 (67%)	0.221
Current	14 (3%)	7 (2%)	7 (2%)	>0.999
Ex-smoker	172 (32%)	106 (32%)	102 (31%)	0.343
BMI				
≥25 kg/m^2^	293 (55%)	200 (60%)	200 (61%)	>0.999
≥30 kg/m^2^	120 (22%)	91 (27%)	92 (28%)	0.789
Hypertension	44 (8%)	33 (10%)	37 (11%)	0.221
Diabetes	10 (2%)	7 (2%)	9 (3%)	0.683
Heart disease	9 (2%)	4 (1%)	4 (1%)	No change
Asthma	101 (19%)	62 (19%)	56 (17%)	0.114
Hospitalised during acute COVID-19	72 (13%)	57 (17%)	57 (17%)	–
Time off work (days)	56 (14, 180)	58 (14, 150)	125 (35, 296)	<0.001
15 common symptoms				
Number reported (median (IQR))	10 (8, 11)	10 (8, 11)	3 (0, 5)	<0.001
None reported in history	0 (0%)	0 (0%)	93 (28%)	<0.001
None reported in history/questionnaires	0 (0%)	0 (0%)	60 (19%)	<0.001
Symptom groups				
Systemic	245 (46%)	159 (48%)	2 (1%)	<0.001
Cardiopulmonary	238 (44%)	143 (43%)	15 (5%)	<0.001
Severe breathlessness (dyspnoea 12 ≥ 10)	187 (36%)	120 (38%)	93 (30%)	0.016
Cognitive dysfunction	268 (50%)	160 (48%)	127 (38%)	0.005
Poor HRQoL	281 (55%)	181 (57%)	138 (45%)	<0.001
Less common symptoms only	66 (13%)	37 (12%)	108 (35%)	<0.001
Duration (days: median, (IQR))				
Initial symptoms-to-assessment	182 (132, 222)	170 (126, 208)	384 (350, 431)	–
COVID-19 positive-to-assessment	110 (53, 175)	110 (53, 170)	328 (265, 375)	–
Organ impairment				
Liver	151 (29%)	119 (36%)	106 (33%)	0.153
Heart	102 (19%)	71 (22%)	70 (21%)	>0.999
Kidney	79 (15%)	60 (18%)	56 (17%)	0.583
Pancreas	100 (20%)	80 (26%)	56 (22%)	0.201
Lungs	12 (2%)	7 (2%)	5 (2%)	>0.999
Spleen	43 (8%)	33 (10%)	29 (9%)	0.453
≥1 organ	314 (59%)	228 (69%)	194 (59%)	<0.001
≥2 organs	122 (23%)	97 (29%)	88 (27%)	0.336

*p*-values represent results from a paired t-test (or non-parametric equivalent), McNemar’s test for categorical and dichotomous variables.

IQR: interquartile range; BMI: body mass index; HRQoL: health-related quality of life.

## Symptoms over 1 year

**Figure 2.**
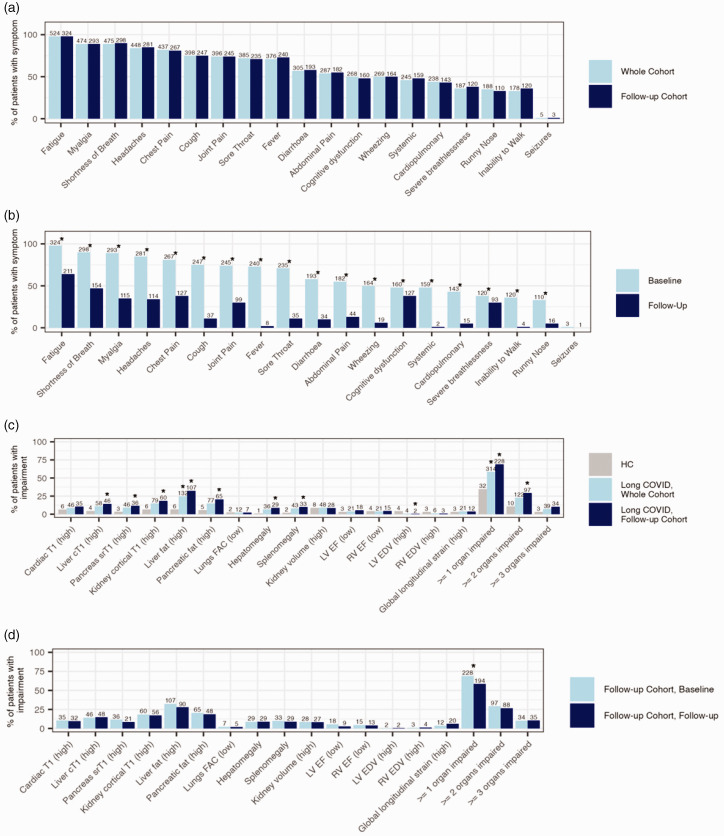
Proportion of individuals with long COVID and symptoms (a: at baseline in the whole cohort and in the follow-up groups; b: at baseline vs. at follow-up in the follow-up group) or impairment (c: in the whole cohort and in the follow-up groups vs. healthy controls; d: at baseline vs. at follow-up in the follow-up group). Significant differences (*p* < 0.05) are indicated with a star, numbers above columns indicate the sample size (*n*) for each group. Note for c: compared between healthy controls (grey) and at baseline for long COVID (blue).

In the whole cohort, at baseline, all participants were symptomatic (Table 1, Figure 2(a)), including those not eligible for follow-up (Figure S2). Females and individuals with obesity were more likely to have ≥1 systemic symptoms, cardiopulmonary symptoms or poor HRQoL (*p* < 0.001, 0.006 and <0.001 for sex and *p* = 0.002, 0.012 and 0.004 for BMI) (Table S4).

In the follow-up group, at baseline and follow-up, participants reported a median of 10 (IQR: 8–11) symptoms and 3 (0–5) symptoms (*p* < 0.001), respectively. At baseline, all five symptom groups had similar prevalence (systemic: 48%, cardiopulmonary: 43%, severe breathlessness: 38%, cognitive dysfunction: 48%, poor HRQoL: 57%); 12% reported none of these symptoms but other less common symptoms instead. At follow-up, symptoms were reduced, particularly systemic (1%) and cardiopulmonary (5%) (*p* < 0.001). Exceptions were fatigue, breathlessness and cognitive dysfunction, where prevalence remained high (Figure 2(b)). Some participants reported these symptoms only at follow-up. Most common symptoms improved by follow-up: fatigue (98% to 64%), myalgia (89% to 35%), shortness of breath (90% to 47%), headache (85% to 34%), chest pain (81% to 38%), fever (73% to 2%), cough (75% to 11%) and sore throat (71% to 11%). Of 331, 60 (18%) had resolved all symptoms at follow-up (Table 2).

**Table 2.** Follow-up group characteristics by symptom status at follow-up.

Characteristic	Ongoing symptomatic (symptoms reported at baseline and follow-up)(*n* = 264)	Resolved symptoms (no symptoms reported at follow-up)(*n* = 60)	*p*-value
Follow-up group baseline characteristics			
Demographics			
Age (years)	46 (10)	48 (12)	0.340
Female sex (*n*)	204 (77%)	34 (57%)	0.002
BMI (kg/m^2^)	26 (23, 30)	28 (23, 31)	0.519
White	239 (91%)	49 (82%)	0.066
Mixed	3 (1%)	0 (0%)	>0.999
South Asian	8 (3%)	7 (12%)	0.010
Black	3 (1%)	2 (3%)	0.232
Healthcare worker	89 (34%)	19 (32%)	0.880
Co-morbidities and risks			
Never smoked	173 (66%)	40 (67%)	>0.999
Current smoker	4 (2%)	2 (3%)	0.308
Ex-smoker	87 (33%)	18 (30%)	0.760
BMI>=25 kg/m^2^	159 (60%)	38 (63%)	0.770
BMI>=30 kg/m^2^	72 (27%)	18 (30%)	0.750
Hypertension	25 (9%)	6 (10%)	0.813
Diabetes	3 (1%)	4 (7%)	0.024
Asthma	53 (20%)	9 (15%)	0.468
Pre-existing heart disease	3 (1%)	1 (2%)	0.561
Hospitalised during acute COVID-19	46 (17%)	10 (17%)	>0.999
Number of common symptoms reported	10 (9, 11)	9 (7, 10)	<0.001
MRI abnormality			
Liver			
cT1 or fat high	95 (36%)	23 (39%)	0.766
cT1 (high)	36 (14%)	9 (15%)	0.836
cT1 (ms)	727 (681, 770)	715 (680, 768)	0.690
Fat (high)	87 (33%)	20 (33%)	>0.999
Fat (%)	3 (2, 6)	3 (1, 6)	0.732
Volume (high)	23 (9%)	6 (10%)	0.801
Volume (ml)	1423 (1252, 1693)	1463 (1277, 1653)	0.650
Pancreas			
srT1 or fat high	64 (26%)	14 (24%)	0.868
srT1 high	29 (12%)	7 (12%)	>0.999
srT1 (ms)	720 (686, 772)	722 (684, 767)	0.775
Fat (high)	51 (20%)	12 (20%)	>0.999
Fat (%)	3 (2, 6)	3 (3, 5)	0.287
Kidney			
Cortex T1 (high)	44 (17%)	14 (24%)	0.193
Cortex T1 left 1.5T (ms)	1084 (74)	1094 (61)	0.323
Cortex T1 right 1.5T (ms)	1072 (70)	1084 (68)	0.260
Cortex T1 left 3T (ms)	1427 (74)	1399 (54)	0.204
Cortex T1 right 3T (ms)	1400 (85)	1382 (70)	0.509
Volume (high)	21 (8%)	7 (12%)	0.318
Volume left (ms)	146 (129, 165)	158 (133, 179)	0.044
Volume right (ms)	146 (128, 166)	150 (137, 172)	0.185
Spleen			
Splenomegaly	28 (11%)	5 (8%)	0.813
Volume (ml)	180 (141, 242)	194 (166, 252)	0.146
Lung			
FAC (low)	7 (3%)	0 (0%)	0.359
FAC (%)	44 (36, 50)	43 (35, 50)	0.844
Heart			
Myocardial Injury/T1 (high)	24 (9%)	11 (18%)	0.062
Average T1 1.5T (ms)	980 (965, 993)	972 (949, 987)	0.047
Average T1 3T (ms)	1182 (31)	1184 (37)	0.903
LV EF (low)	13 (5%)	4 (7%)	0.531
LV EF (%)	60 (5)	58 (5)	0.029
RV EF (low)	10 (4%)	4 (7%)	0.302
RV EF (%)	59 (5)	58 (6)	0.108
LV EDV (high)	2 (1%)	0 (0%)	>0.999
LV EDV (ml)	78 (71, 87)	82 (75, 94)	0.008
RV EDV (high)	3 (1%)	0 (0%)	>0.999
RV EDF (ml)	75 (66, 84)	80 (70, 93)	0.005
Global longitudinal strain (high)	11 (4%)	0 (0%)	0.230
Global longitudinal strain (%)	−14 (2)	−14 (2)	0.474
Number of organs impaired on MRI			
0	84 (32%)	17 (28%)	0.646
≥1	180 (68%)	43 (72%)	0.646
≥2	75 (28%)	20 (33%)	0.438
≥3	26 (10%)	7 (12%)	0.641
Follow-up characteristics			
Hospitalised between baseline and follow-up	13 (5%)	1 (2%)	0.480
Time off work (days)	172 (42, 300)	54 (21, 120)	<0.001
MRI abnormality			
Liver			
cT1 or fat high	88 (35%)	17 (28%)	0.447
cT1 (high)	40 (16%)	7 (12%)	0.547
cT1 (ms)	724 (693, 768)	708 (676, 755)	0.150
Fat (high)	74 (29%)	16 (27%)	0.754
Fat (%)	3 (2, 6)	3 (2, 6)	0.877
Volume (high)	24 (9%)	5 (8%)	>0.999
Volume (ml)	1427 (1271, 1699)	1474 (1316, 1675)	0.429
Pancreas			
srT1 or fat high	43 (22%)	10 (21%)	>0.999
cT1 high	16 (8%)	4 (8%)	>0.999
srT1 (ms)	720 (684, 754)	700 (673, 740)	0.113
Fat (high)	35 (18%)	10 (20%)	0.683
Fat (%)	3 (2, 5)	3 (2, 6)	0.989
Kidney			
Cortex T1 (high)	45 (17%)	9 (15%)	0.848
Cortex T1 left 1.5T (ms)	1086 (58)	1086 (67)	0.949
Cortex T1 right 1.5T (ms)	1082 (53)	1078 (67)	0.702
Cortex T1 left 3T (ms)	1422 (91)	1424 (53)	0.931
Cortex T1 right 3T (ms)	1404 (85)	1393 (59)	0.605
Volume (high)	21 (8%)	6 (10%)	0.610
Volume left (ms)	145 (127, 165)	158 (137, 180)	0.011
Volume right (ms)	149 (129, 167)	156 (143, 172)	0.028
Spleen			
Splenomegaly	24 (9%)	5 (8%)	>0.999
Volume (ml)	176 (139, 246)	192 (161, 245)	0.074
Lung			
FAC (low)	4 (2%)	0 (0%)	0.580
FAC (%)	45 (38, 52)	45 (38, 52)	0.752
Heart			
Myocardial Injury / T1 (high)	25 (9%)	7 (12%)	0.632
Average T1 1.5T (ms)	982 (969, 992)	975 (960, 987)	0.069
Average T1 3T (ms)	1182 (38)	1184 (48)	0.895
LV EF (low)	6 (2%)	2 (3%)	0.644
LV EF (%)	60 (4)	58 (5)	0.013
RV EF (low)	7 (3%)	5 (8%)	0.051
RV EF (%)	59 (5)	57 (5)	0.014
LV EDV (high)	2 (1%)	0 (0%)	>0.999
LV EDF (ml)	78 (70, 86)	82 (75, 92)	0.012
RV EDV (high)	4 (2%)	0 (0%)	>0.999
RV EDF (ml)	74 (66, 86)	81 (72, 91)	0.015
Global longitudinal strain (high)	15 (6%)	5 (8%)	0.551
Global longitudinal strain (%)	−15 (2)	−14 (2)	0.017
Number of organs impaired on MRI			
0	112 (42%)	23 (38%)	0.664
≥1	152 (58%)	37 (62%)	0.664
≥2	73 (28%)	13 (22%)	0.419
≥3	28 (11%)	5 (8%)	0.813

Compared are the baseline characteristics (top part) and the follow-up characteristics (bottom part) between groups that at follow-up were ongoing symptomatic or had resolved symptoms. Values in red are p < 0.05.

## Function over 1 year

HRQoL was poor at baseline in the whole cohort of individuals with long COVID: median visual analog score (VAS) score 60% (IQR: 40%–70%) and median reported health utility index score 0.67 (IQR: 0.48–0.77). The most highly ranked sub-optimal health dimensions were problems completing usual activities of living and pain (56% and 45%, respectively, reporting moderate to extreme difficulties).

These difficulties were also observed at baseline in the follow-up group (median VAS score 60%, IQR: 40%–70%, and median reported health utility index score of 0.67, IQR: 0.48–0.77) (Figures S3 and S4). At follow-up, there was increased health utility index score to 0.71 (range 0.56–0.81) (*p* < 0.001), but 42% still reported utility score <0.7, and 28% still complained of severe breathlessness. Almost every individual at follow-up (271/302, 90%) had taken COVID-19-related time off work (median: 125 days, IQR: 35–296). At follow-up, 95% of healthcare workers had taken time off work (median 180 days, range 41–308) and 62 (63%) had taken >100 days off work. Those with ongoing symptoms reported taking more time off work (median 172 days, range 42–300) compared to those whose symptoms had resolved (median 54 days, range 21–120) (Table 2).

## Organ impairment over 1 year

Most standard-of-care biochemical investigations were within normal range and not predictive of outcomes (Table S5), except lactate dehydrogenase (59/306 (19%) and 70/319 (22%)), creatinine kinase (26/313 (8%) and 41/323 (13%)); cholesterol (152/313 (49%) and 157/326 (48%)); and mean cell haemoglobin concentration (62/313 (20%) and 49/324 (15%); *p* = 0.05), which were elevated at both baseline and follow-up.

On MRI of 536 participants at baseline, 59% and 23% had impairment in ≥1 and ≥2 organs, respectively (Figure 2(c)), although impairment was usually mild (Table S6), e.g. among participants with cardiac impairment, none had severe heart failure. Liver steatosis, kidney fibro-inflammation and splenomegaly at baseline were more frequent in all symptom groups (Table S4). Liver steatosis was associated with systemic symptoms and severe breathlessness (Figure S5).

In the follow-up group, single-organ impairment between visits improved but remained high (69% at baseline and 59% at follow-up had impairment in ≥1 organ; *p* < 0.001) without improvement in individual metrics between visits (Figure 2D). Multi-organ impairment did not improve (29% to 27%; *p* = 0.336). Individuals without organ impairment had lower symptom burden compared to those with at least one organ impairment (Figure S6). At baseline, lung impairment (lower fractional area change) and impairment in ≥3 organs had the highest symptom burden. Healthcare workers were more likely to have liver impairment (*p* = 0.015 at baseline and *p* = 0.034 at follow-up) than the rest of the cohort.

In individuals without symptoms at follow-up (*n* = 60), organ impairment was present in 43 (72%) at baseline and 37 (62%) at follow-up (Table 2, Figure S6). In symptomatic participants at follow-up (*n* = 264), there were 84 (32%) and 112 (42%) individuals without organ impairment at baseline and follow-up, respectively.

## Associations between symptoms and organ impairment

**Figure 3.**
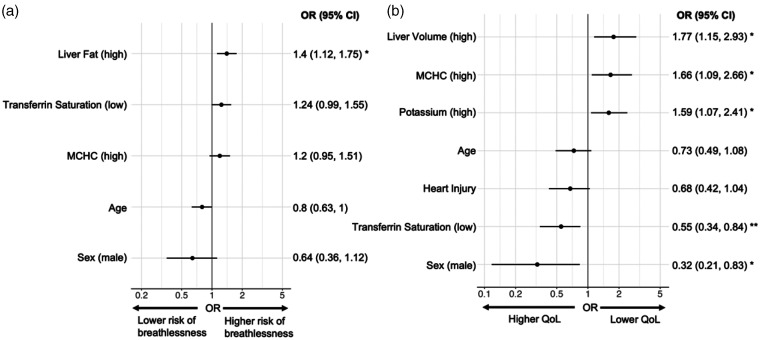
Association between risk factors and: (a) severe breathlessness at baseline; (b) poor HRQoL at follow-up. Note: (a) associations predicted whole cohort symptom at baseline using baseline covariates and (b) predicted follow-up symptom using follow-up covariates in the follow-up group.

Looking at individual symptoms and by five symptom groups, neither abnormal biochemical investigations nor organ impairment were predictive of full symptom resolution at follow-up (Tables S4–S7). Several liver-specific parameters were associated with specific symptom burden (Figure 3, Table S7). High liver fat was present in 58/187 with severe breathlessness but only 70/328 without severe breathlessness at baseline. Conversely, low liver fat was more likely in those without severe breathlessness, at both timepoints, in the follow-up group. High liver volume at follow-up was associated with lower HRQoL. Hepatomegaly was present in 20/138 with poor QoL but in only 8/167 with better QoL at follow-up.

## Discussion

In this UK prospective study of largely non-hospitalised long COVID, we have four new findings. First, we confirm multi-organ impairment at 6 and 12 months in 29% of individuals with long COVID, with persistent symptoms and reduced function. Second, despite some associations between organ impairment and symptoms, there is currently insufficient evidence for distinct long COVID subtypes. Blood biomarkers, the current standard of care, showed no relation to clinical presentation. Third, symptoms, blood investigations and quantitative, multi-organ MRI did not predict trajectory or recovery. Fourth, we demonstrate feasibility of scalable, multi-organ assessment in non-acute settings in the pandemic context.

Several studies confirm persistence of symptoms in individuals with long COVID up to 1 year.^16^ We now add that three in five people with long COVID have impairment in at least one organ, and one in four have impairment in two or more organs, in some cases without symptoms. Impact on quality of life and time off work, particularly in healthcare workers, is a major concern for individuals, health systems and economies.^17^ Many healthcare workers had no prior illness (2% diabetes, 2% heart disease and 22% asthma, which may play a pathophysiologic role^18^) but of 172 such participants, 19 were still symptomatic at follow-up and off work for a median of 180 days. We need comparison with similar analyses from other long COVID studies, and other long-term conditions,^19^ with considerable workforce planning implications. There have been heterogenous methods to investigate long COVID, whether qualitative^20,21^ or quantitative,^5^ or symptom surveys^6^ versus cohort studies.^4,22^ Most research still focuses on individual organs.^23–25^ The scale of long COVID burden necessitates action to develop, evaluate and implement evidence-based investigation, treatment and rehabilitation^26^ (e.g. STIMULATE-ICP and other studies^4^).

Metabolic diseases, including non-alcoholic liver disease and diabetes, are postulated to play a role in the pathogenesis of severe COVID-19 and possibly long COVID.^27^ We observe associations with markers of liver dysfunction, and increased risk of symptoms in female and obese individuals with long COVID, which have been shown previously.^28,29^ Cognitive impairment appears to develop in the natural history of long COVID in some patients. However, the underlying mechanisms of the condition or syndrome remain elusive. We did not find evidence by symptoms, blood investigations or MRI to clearly define long COVID subtypes.

We observed symptom resolution at 12 months in 29% of individuals with long COVID, particularly with cardiopulmonary and systemic symptoms, aligning with other longitudinal studies.^5,13,19^ Our findings of reductions in HRQoL and time at work reinforce prior research and are concerning, despite improvement over time. Future research must consider associations between symptoms, multi-organ impairment and function in larger cohorts, enabling clearer stratification and evaluation of treatments.

 The COVERSCAN study was initiated in the early COVID-19 pandemic’s first wave when face-to-face assessment and investigation, and reduced health system capacity were major concerns for patients and health professionals. In the UK and other countries, long COVID carries high burden of investigations and healthcare utilisation across specialties, and definitive care pathways are lacking. We show feasibility, acceptability and scalability of a rapid (40-min), multi-organ MRI protocol for practice and research. Alongside routine clinical assessment and blood tests, COVERSCAN can exclude organ impairment in integrated, multidisciplinary care pathways.^30^ Such MRI assessment has potential application beyond the pandemic for multi-system assessment and investigation, including in lower resource settings.

## Implications for research

There are three research implications. First, complex intervention trials, including care pathways from investigation to rehabilitation are needed to evaluate therapies. Second, associations between symptoms, symptom groups, blood investigations and MRI must be investigated in larger populations. Third, long COVID pathophysiology is still unclear, and should ideally be studied at the same time as clinical trials.

## Implications for clinical practice and public health

There are three practice and policy implications. First, COVERSCAN could be used to rule out organ impairment and to identify subgroups requiring specialist referral. Second, long COVID is a multi-organ condition, needing multi-organ assessment and multidisciplinary care. Third, poor 1-year post-COVID recovery rates highlight the need for rehabilitation and integrated care, relevant to other long-term conditions.

## Strengths and limitations

To date, this is the largest, comprehensive, systematic, multi-organ, post-COVID study over 1 year. The study cohort is representative of long COVID resulting from the first and second waves in the UK in a mainly community setting in terms of risk factors and acute COVID-19 hospitalisation.^7,8^ There are limitations. We acknowledge that the study may not be representative of the condition in patients infected with other variants of SARS-CoV-2, or of long COVID in mainly hospitalised patients. Our study recruited patients who self-referred from online patient support forums and social media rather than a systematic screen of post-COVID patients, as long COVID clinics were not yet established. We did not have history and imaging prior to the pandemic and so it is difficult to determine if COVID-19/long COVID caused impairment. Despite low prevalence of self-reported co-morbidities in this cohort, in some participants the organ impairment we identified may be due to pre-existing, undiagnosed co-morbidities rather than direct post-COVID sequelae. There was no assessment of brain function. Not all participants had a laboratory-confirmed COVID-19 diagnosis. Generalisability of results from the UK’s first COVID-wave to the global population requires further exploration. We successfully delivered multi-organ MRI to assess organ health, but clinical utility remains to be determined. Health utilisation (e.g. primary care interactions) and economic burden of long COVID were not assessed. Time off work was not assessed at baseline when the UK was in lockdown with a national furlough scheme in operation. Patients with normal assessment at baseline were not followed up to minimise in-person contact and due to resource and funding constraints. Therefore, while 222/536 (41%) patients at baseline were defined as normal after assessment, we cannot say that they had better outcomes, and this may represent a selection bias. Nevertheless, we can model cost-saving of a single assessment versus multiple assessments to achieve a discharge decision.

## Conclusions

Long COVID symptoms commonly persist at 12 months, even in those not severely affected by acute COVID-19. Diagnosis and follow-up of long COVID can be performed in non-acute settings. Continued research in multi-system assessment and pharmacotherapy for those reporting ongoing fatigue, breathlessness and cognitive problems is required to address long COVID burden, in parallel with mechanistic studies to understand pathophysiology.

## Key points

- Question: What is the prevalence of organ impairment in long COVID at 6 and 12 months post COVID-19?
- Findings: In a prospective study of 536 mainly non-hospitalised individuals, all were symptomatic at 6 months and 59% had single-organ impairment. Although symptom burden decreased, organ impairment persisted in the 331 followed up at 12 months post COVID-19.
- Meaning: Organ impairment in long COVID has implications for symptoms, quality of life and longer-term health, signalling the need for prevention and integrated care of long COVID.

## Supplemental Material

sj-pdf-1-jrs-10.1177_01410768231154703 - Supplemental material for Multi-organ impairment and long COVID: a 1-year prospective, longitudinal cohort study Click here for additional data file. Supplemental material, sj-pdf-1-jrs-10.1177_01410768231154703 for Multi-organ impairment and long COVID: a 1-year prospective, longitudinal cohort study by Andrea Dennis, Daniel J Cuthbertson, Dan Wootton, Michael Crooks, Mark Gabbay, Nicole Eichert, Sofia Mouchti, Michele Pansini, Adriana Roca-Fernandez, Helena Thomaides-Brears, Matt Kelly, Matthew Robson, Lyth Hishmeh, Emily Attree, Melissa Heightman, Rajarshi Banerjee and Amitava Banerjee in Journal of the Royal Society of Medicine

## Declarations

### Guarantor

AB.

### Provenance

Not commissioned.

### Availability of data and materials

All relevant data to the study are included in the article and the supplementary material.

### ORCID iD

Amitava Banerjee https://orcid.org/0000-0001-8741-3411
