# Supplementary material for: Multi-organ impairment and long COVID: a 1-year prospective, longitudinal cohort study
Source: J R Soc Med. 2023 Feb 14;116(3):97–112. doi: 10.1177/01410768231154703 (PMC10041626; doi:10.1177/01410768231154703)
Supplement: sj-pdf-1-jrs-10.1177_01410768231154703 - Supplemental material for Multi-organ impairment and long COVID: a 1-year prospective, longitudinal cohort study [file sj-pdf-1-jrs-10.1177_01410768231154703.pdf]

**Page Number**	**Contents**
2	Supplementary Methods
5	Table S1a: Demographics at baseline for those recovering from COVID-19 (whole cohort) vs the controls used to derive reference ranges (median [IQR] or count [%]).
6	Table S1b: Reference ranges for MRI metrics.
8	Table S2: Differences in organ impairment and symptoms by COVID-19 diagnosis method for the whole cohort at baseline
10	Table S3: Baseline characteristics of individuals with organ impairment identified at baseline, eligible for follow-up (the follow-up group) vs those without impairment, who were only invited for the baseline visit
11	Table S4: Characteristics for individuals with long COVID (whole cohort) at baseline, comparing those with common symptoms (systemic, cardiopulmonary, severe breathlessness, cognitive dysfunction, poor health related quality of life) and those with less common symptoms.
13	Table S5: Blood investigations in the whole cohort and the follow-up group of individuals with long Covid.
15	Table S6. Comparison of MRI metrics between healthy controls and whole long COVID cohort at baseline.
16	Table S7: Associations between biomarkers and symptom groups in individuals with long COVID.
18	Figure S1: Example MRI data segmentations used in organ morphology measurements.
19	Figure S2: Symptoms at baseline in long COVID individuals with organ impairment identified at baseline, eligible for follow-up (the follow-up group) vs those without impairment, who were only invited for the baseline visit
20	Figure S3: Self-reported health related quality of life as reported from the EQ-5D-5L instrument showing the UK specific index scores (left) and the visual analogue score (right), in the follow-up group
21	Figure S4: Dimensions of health from the EQ-5D-5L questionnaire in the follow-up group.
22	Figure S5: Proportion of long COVID cases with liver steatosis by symptom group (systemic, cardiopulmonary, severe breathlessness, brain fog, poor HRQoL).
23	Figure S6: Heat maps showing the proportion of those with impairment in individual organs that reported specific symptoms at baseline (left) and follow-up (right) in the follow-up group
24	Supplementary references

Supplementary Methods

Inclusion and exclusion criteria

Inclusion criteria were recent confirmed diagnosis of SARS-CoV-2 RNA via a polymerase chain reaction (PCR) assay (having been discharged 7 or more days from hospital, where hospital admission was required), a positive SARS-CoV-2 antibody test or clinically suspected COVID-19 by at least two qualified clinicians. Exclusion criteria were contraindications to MRI (e.g. pacemakers, defibrillators, metallic implanted devices, claustrophobia) and any of: active respiratory infection symptoms (temperature >37.8°C or ≥3 coughing episodes in 24 hours); hospital discharge in last 7 days; asymptomatic for ≥4 months prior to enrolment, or hospital discharge ≥4 months prior to enrolment (to harmonise time post-infection across the study).

SARS-CoV-2 Diagnosis

Prior SARS-CoV-2 infection was defined based on a laboratory-confirmed tests (positive oro-/nasopharyngeal SARS-CoV-2 swab test by reverse-transcriptase-polymerase-chain reaction, **n=151,** or antibody test**, n=145**). Furthermore, strong clinical suspicion of infection (typical symptoms and COVID-19 diagnosis by two independent clinicians) was included as criterion **(n=240)**, as testing was not widely available to non-hospitalized patients at the time of study recruitment. In n=18 patients, clinical diagnosis was performed only by one clinician (clinical equipoise).

Study recruitment

Participants were informed about the study via promotional material, disseminated via health authority centers, patient online forums and websites. The study website (coverscan.com) was advertised by Perspectum or by iWGC (an independent platform and service provider for patient experience data collection and analysis) via online posting (social media, website banners) to the general public and by global invitation emails to existing networks of patients consented to hear of new research.
Participants interested in the study registered their interest by responding to a link located on the study's webpage. The majority of participants heard of the study from social media (350/536) or by word of mouth (122/536), while few responded to advertising (42/536) or came from hospital referrals (16/536). All self-referred participants underwent systematic screening for eligibility before enrolment into the study, conducted by trained clinical research personnel using a standardized questionnaire. Participation was voluntary (no remuneration other than small contribution to travel costs).

Symptom collection and classification

Self-reported symptoms for recruited participants in the study were collected through several means: 1) through responses to validated questionnaires (EQ-5D-5L/Dyspnoea-12) and 2) as part of medical history collection. From medical history 15 common, self-reported symptoms were identified: history of fever, cough, sore throat, runny nose, wheezing, chest pain, myalgia, joint pain, fatigue or malaise, shortness of breath, inability to walk, headaches, seizures, abdominal pain, diarrhoea. HRQoL was assessed by validated EQ-5D-5L (EuroQOL), comprising: (1) five health dimensions (mobility, self-care, usual activities, pain and discomfort, and anxiety and depression) each at five severity levels (none, slight, moderate, severe, and extreme); (2) self-rated health using visual analogue scale (VAS) from 0 (worst imaginable) to 100 (best imaginable); (3) derived EQ-5D 'utility' or index score from 0 ('dead') to 1 ('full health').
The most common symptoms were classified according to published subgroups[9]: systemic (fever, myalgia, joint pain, fatigue/malaise, headaches), cardiopulmonary (wheezing, chest pain, shortness of breath), cognitive dysfunction (poor memory, cognition and concentration), poor HRQoL (EQ-5D utility score<0.7) and a subset of the cardiopulmonary subgroup for severe breathlessness (dyspnoea-12 total score≥10). Participants who only reported more unusual, less common, symptoms (such as sensitivity to light, hallucinations, rib pain), which did not clearly fit into these categories, were defined as having "less common symptoms only").

Imaging Acquisitions

•	Cardiac MR imaging involved complete coverage of the heart with a short-axis stack (from the apex to the valve plane) of cine images acquired using cardiac gating, each acquired within a short breath-hold. This acquisition mirrors the one used at the UK Biobank and is a standardized approach [1]. Three short-axis and two long axis cine (HLA and VLA) cardiac T1 maps were also acquired using the MOLLI-T1 approach at the basal, mid, and apical levels of the left ventricle. Three short-axis and two long axis cine (HLA and VLA) at the basal, mid, and apical levels. For cardiac T2 maps, acquired using the fast low angle shot (FLASH) in 3T, at 1.5T scanner, TrueFISP was used (a Siemens version of balanced SSFP).
•	Liver and pancreas imaging used the LiverMultiScan acquisition protocol (Perspectum, Oxford, UK), which involves 3 single 2D axial slice breath-held acquisitions that separately are sensitive to the fat content (proton density fat fraction [PDFF]), to T2* (which can yield liver iron content) and a MOLLI-T1 measurement (providing a measurement of tissue water).Additionally, a volumetric scan was used that covers the entire liver [2].
•	Lungs: Two single slice dynamic cine MR acquisitions were acquired in the coronal plane with a 307ms temporal resolution: one 40 s acquisition with the patient instructed to breathe normally and a second 30 s acquisition with the patient instructed to breathe deeply.
•	Kidney: a single coronal view was used to image both kidneys. Imaging contrasts were MOLLI-T1, and a spoiled gradient recalled acquisition (spGR).
•	Spleen: Volumetric spGR MRI images

Image Analysis
•	Cardiac: Experienced cardiac MRI analysts used CVI42v5.11 (Cardiovascular Imaging Inc, Canada) to trace manually the myocardium in the end-diastolic and end-systolic phases in each of the short-axis views, following the standard UK Biobank evaluation approach as previously described [3]. We reported ventricular function; end systolic and diastolic volume; stroke volume and ejection fraction in both ventricles; left ventricular muscle mass and ventricular max wall thickness and global longitudinal and circumferential 3D strain metrics. Cardiac T1 and T2 measurements were determined as the mean of each of the 16 cardiac segments (of the AHA 17 segment model excluding the apex) [4]. When artefacts were present those measurements were not included in the results.
•	Liver Images were analysed by data analysts experienced at using the LiverMultiScan (Perspectum, Oxford, UK) software. This yielded global metrics in each liver of PDFF (proton density fat fraction), T2*, and cT1 (cT1 is a measurement of T1 that has been corrected for the confounding effects of iron and standardised to 3 Tesla; it is elevated with disease).
•	Pancreas images were analysed in an equivalent manner to the above except the software used was not FDA-cleared and iron correction was not performed [5]. The T1 was standardized to 3 Tesla.
•	Lung: Patient respiration was assessed by imaging a single 2D coronal slice of the lungs using a 30 second dynamic cine MRI acquisition (with a time resolution of 307ms), during which the patient was instructed to breathe deeply. In-house developed automated segmentation methods were used to segment the lungs and measure their areas in each time frame. Segmentations were reviewed and corrected by trained analysts. From these area vs time measurements, the lung area at maximum inspiration and expiration was measured, as well as a fractional area change (max area - min area)/max area.
•	Kidney: assessed using in-house tools to fit parametric maps and to allow trained analysts to make measurements. The kidney cortex was manually segmented using the MOLLI-T1 map to guide the boundary. Multiple regions-of-interests were manually placed within the cortex to extract a median value of cortical T1 in each kidney. Volumetric delineations of the kidneys were derived from SPGR MRI images. Automated delineations were produced using a 3D convolutional neural network, trained on expert annotations. Delineations were manually checked, and corrected, if necessary, for each subject.
•	Spleen: Volumetric delineations were derived from SPGR MRI images. Automated delineations were produced using a 3D convolutional neural network, trained on expert annotations. Delineations were manually checked, and corrected, if necessary, for each subject.

Technical failures were considered to be those acquisitions which did not produce images of sufficient quality as to derive metrics reported herein. For cardiac T1 and T2, this was based on derivation of global values for 3 segments.

Reference Ranges

All MRI metric reference ranges, but organ volumes, were calculated with n=92 Healthy Controls (HC) scanned at 1.5T and 3T based on the 2.5% (lower threshold) and 97.5% percentiles (upper threshold), using bootstrapping (100,000 permutations). Organ volumes were calculated from a combined cohort of 92 healthy controls and 1744 BMI-matched participants from the UK Biobank[6], representing all sex and height subgroups, as these are known confounders of organ size [7].

Analysis

Organ Impairment

Organ impairment was calculated for each organ based on evidence of any of the measurement appearing out of reference range (*Liver:* elevated cT1 or Fat; *Kidney:* elevated T1; *Pancreas:* elevated srT1 or Fat; *Heart:* elevated T1 in 3 or more segments, decreased RV or LV EF or increased LV or RV EDV or increased LV global longitudinal strain; *Spleen*: elevated volume; *Lung:* reduced fractional area volume). Thus, depending on the metric the upper or lower threshold was considered. For cardiac T1, elevation in at least 3 AHA segments defined elevated T1. Single organ impairment was based on at least 1 organ impaired and multi-organ impairment was based on 2 organ impairments. Elevations in liver or kidney volume were excluded from definitions of organ impairment, as these were considered anatomical findings subject to diurnal variation[20] but not necessarily hallmarks of impairment and specific histological features of disease[11]. Since pre-COVID data were unavailable, organ impairment may pre-date COVID-19. A sensitivity analysis was conducted, excluding those with metabolic syndrome risk factors on study entry, in whom organ impairment is more likely to have pre-existed before first symptoms of COVID-19 (including BMI ≥30 kg/m2, hypertension and diabetes).

Associations of Symptoms and Biomarkers

For multivariable regression, the outcomes of interest included separately the most common symptom groups systemic (reporting: fever, myalgia, joint pain, fatigue/malaise, headaches), cardiopulmonary (wheezing, chest pain, shortness of breath), severe breathlessness (dyspnoea-12 total score >=10), cognitive dysfunction (reporting any problems with memory, cognition and concentration), and poor HRQoL (EQ-5D utility score <0.7).

The regressors included i) demographic characteristics (age, sex, and BMI); ii) elevation in T1 metrics, steatosis or organ volume defined as a binary variable (liver cT1, PDFF and volume; pancreas srT1 and PDFF; kidney cortex T1 and volume, spleen volume, lung FAC, heart injury); iii) blood investigations outside of normal range in 15% of participants or significantly different between visits (cholesterol, HDL cholesterol, lactate dehydrogenase [LDH], mean corpuscular haemoglobin concentration [MCHC], potassium, low transferrin saturation, creatine kinase, basophils, low total iron binding capacity [TIBC], C-peptide) defined as binary variables. All continuous variables (BMI and age) were z-scored before entering the statistical models. Only observations with no missing data were included in the models.

For each outcome, first the effect of organ impairment was assessed in a multi-variable stepwise regression model, including regressors for demographics and all MRI organ metrics. Second, the effect of blood markers was assessed in a separate multi-variable stepwise regression model including demographic factors. Third, we performed a final multi-variable stepwise regression including regressors for demographic characteristics and both the MRI and blood metrics combined. Standardized odd ratios (OR) with 95% confidence intervals from this regression were presented in forest plots.

Table S1a: Demographics at baseline for those recovering from COVID-19 (whole cohort) vs the controls used to derive reference ranges (median [IQR] or count [%]).

	**Long COVID n=536**	**control group 1, n=92**	**p-value***	**control group 2, n=1835**	**p-value****
Sex (n female)	389 (73%)	61 (66%)	0.3	1024 (56%)	**<0.001**
Age	44 (38, 52)	44 (32, 53)	0.5	56 (49, 61)	**<0.001**
BMI	26 (23, 29)	23 (21, 25)	**<0.001**	25 (23, 28) (missing: n=3)	0.2
Ethnicity			0.093		**<0.001**
White	477 (89%)	85 (92%)		1794 (98%)	
Black	13 (2%)	0 (0%)		5 (0%)	
Chinese	2 (0%)	2 (2%)		7 (0%)	
South Asian	22 (4%)	16 (1%)		13 (1%)	
Other	22 (4%)	1 (1%)		16 (1%)	
Smoking status				(missing: n=3)	**0.021**
Current smoker	14 (3%)	3 (3%)	0.7	101 (6%)	
Past smoker	172 (32%)	17 (18%)	**0.012**	591 (32%)	
Hypertension	44 (8%)	0 (0%)	**0.009**	0 (0%)	**<0.001**
Diabetic	10 (2%)	0 (0%)	0.4	0 (0%)	**<0.001**
Heart disease	9 (2%)	0 (0%)	0.21	65 (4%) (missing: n=346)	**0.014**
Asthma	101 (19%)	0 (0%)	**<0.001**	318 (23%) (missing: n=435)	**<0.001**

*p-value of comparisons between long COVID patients and controls for reference ranges for all metrics but organ volumes (control group 1, n=92). **p-value of comparisons between long COVID participants and a larger pool of healthy controls for organ volumes (control group 2, n=1836).

Table S1b: Reference ranges for MRI metrics.

	**Sex**	**Field Strength**	**Height (cm)**	**Lower threshold**	**Upper threshold (*)**
CARDIAC METRICS					
Field strength independent metrics (body-surface area [BSA] corrected)					
Left end diastolic volume (ml)	F	-	-	-	108
Left end diastolic volume (ml)	M	-	-	-	132
Right end diastolic volume (ml)	F	-	-	-	110
Right end diastolic volume (ml)	M	-	-	-	139
Field strength independent metrics (not BSA corrected)					
Global longitudinal strain 3D (%)	F	-	-	-	-11.45
Global longitudinal strain 3D (%)	M	-	-	-	-7.75
Left ventricle ejection fraction (%)	F	-	-	52	-
Left ventricle ejection fraction (%)	M	-	-	51	-
Right ventricle ejection fraction (%)	F	-	-	50	-
Right ventricle ejection fraction (%)	M	-	-	50	-
Field strength dependent metrics					
Global T1 ref range (ms) (#)	F	1.5T	-	-	1042
Global T1 ref range (ms) (#)	M	1.5T	-	-	997
Global T2 ref range (ms) (#)	-	1.5T	-	-	51
Global T1 ref range (ms) (#)	F	3T	-	-	1255
Global T1 ref range (ms) (#)	M	3T	-	-	1214
Global T2 ref range (ms) (#)	-	3T	-	-	46
LIVER METRICS					
Field strength independent metrics					
cT1 (ms)	-	-	-	-	800 (*)
PDFF (%)	-	-	-	-	5 (*)
Volume (ml)	F	-	< 164	-	1778
Volume (ml)	M	-	< 164	-	2003
Volume (ml)	F	-	≥ 164<br>< 250	-	2048
Volume (ml)	M	-	≥ 164<br>< 250	-	2284
KIDNEY METRICS					
Field strength independent metrics					
Left volume (ml)	F	-	< 164	-	177
Left volume (ml)	M	-	< 164	-	221
Left volume (ml)	F	-	≥ 164<br>< 250	-	192
Left volume (ml)	M	-	≥ 164<br>< 250	-	255
Right volume (ml)	F	-	< 164	-	176
Right volume (ml)	M	-	< 164	-	207
Right volume (ml)	F	-	≥ 164<br>< 250	-	186
Right volume (ml)	M	-	≥ 164<br>< 250	-	229
Field strength dependent metrics					
Left or Right Cortical T1 (ms) (§)	-	1.5T	-	-	1154
Left or Right Cortical T1 (ms) (§)	-	3T	-	-	1512
PANCREAS METRICS					
Field strength independent metrics					
srT1 (ms)	-	-	-	-	821
PDFF (%)	-	-	-	-	6.6 (*)
SPLEEN METRICS					
Field strength independent metrics					
Volume (ml)	F	-	< 164	-	255

	**Sex**	**Field Strength**	**Height (cm)**	**Lower threshold**	**Upper threshold (*)**
Volume (ml)	M	-	< 164	-	392
Volume (ml)	F	-	≥ 164<br>< 250	-	293
Volume (ml)	M	-	≥ 164<br>< 250	-	411
LUNG METRICS					
Field strength independent metrics					
Total deep fractional area change (%)	-	-	-	22.0	-

(*) Reference ranges for the liver cT1 and liver PDFF were established from literature [8]. For pancreas PDFF, which has a positive skew in the distribution, reference ranges were extracted with the 95% percentile. (§) Right and left kidney cortex T1 limits were averaged for threshold setting. (#) Cardiac T1 and cardiac T2 were measured for each of 16 AHA segments but thresholds are reported for the global average.

Table S2: Differences in organ impairment and symptoms by COVID-19 diagnosis method for the whole cohort at baseline. P-values represent results from an independent two-sample t-test (or non-parametric equivalent), Fisher's exact test for dichotomous variables and for categories.

	**Whole cohort**		
**Characteristic**	**Clinically diagnosed COVID-19 (n=240)**	**COVID-19 positive test result (n=296)**	**P Value**
Age (years)	44 (9)	45 (11)	0.074
Sex (n females)	184 (77%)	205 (69%)	0.064
BMI (kg/m^2)	25 (22, 28)	26 (23, 30)	**0.003**
Ethnicity			0.085
White	221 (92%)	256 (86%)	
Mixed	10 (4%)	11 (4%)	
South Asian	6 (2%)	18 (6%)	
Black	3 (1%)	10 (3%)	
Healthcare worker	46 (19%)	126 (43%)	**<0.001**
At least one COVID-19 vaccination	3 (1%)	7 (2%)	0.524
**Comorbidities and risks**			
Smoking			0.441
Never	149 (62%)	200 (68%)	
Current	7 (3%)	7 (2%)	
Ex-smoker	83 (35%)	89 (30%)	
BMI			
≥25 kg/m^2	109 (45%)	184 (62%)	**<0.001**
≥30 kg/m^2	44 (18%)	76 (26%)	**0.048**
Hypertension	19 (8%)	25 (8%)	0.875
Diabetes	4 (2%)	6 (2%)	>0.999
Heart disease	5 (2%)	4 (1%)	0.523
Asthma	49 (20%)	52 (18%)	0.437
Hospitalised during acute COVID-19	25 (10%)	47 (16%)	0.075
Time off work (days)	180 (150, 300)	30 (12, 120)	**<0.001**
**15 common symptoms**			
Number reported [median (IQR)]	10 (9, 12)	9 (8, 11)	**<0.001**
None reported in history	0 (0%)	0 (0%)	No Change
None reported in history/ questionnaires	0 (0%)	0 (0%)	No Change
**Symptom groups**			
Systemic	120 (50%)	125 (42%)	0.081
Cardiopulmonary	130 (54%)	108 (36%)	**<0.001**
Severe breathlessness (Dyspnoea 12 ≥10)	104 (45%)	83 (29%)	**<0.001**
Cognitive dysfunction	135 (56%)	133 (45%)	**0.012**
Poor HRQoL	156 (67%)	125 (44%)	**<0.001**
Less common symptoms only	15 (6%)	51 (18%)	**<0.001**
**Duration (days: median, [IQR])**			
Initial symptoms-to-assessment	195 (162, 227)	160 (106, 215)	**<0.001**

	**Whole cohort**		
**Characteristic**	**Clinically diagnosed COVID-19 (n=240)**	**COVID-19 positive test result (n=296)**	**P Value**
COVID-19 positive-to-assessment	134 (60, 187)	97 (53, 153)	**0.002**
**Organ Impairment**			
Liver	53 (23%)	98 (34%)	**0.007**
Heart	43 (18%)	59 (20%)	0.580
Kidney	38 (16%)	41 (14%)	0.624
Pancreas	41 (18%)	59 (21%)	0.432
Lungs	6 (3%)	6 (2%)	0.776
Spleen	21 (9%)	22 (7%)	0.633
≥1 organ	132 (55%)	182 (61%)	0.135
≥2 organs	48 (20%)	74 (25%)	0.179

Table S3: Baseline characteristics of individuals with organ impairment identified at baseline, eligible for follow-up vs those without impairment, who were only invited for the baseline visit. P-values represent results from an independent two-sample t-test (or non-parametric equivalent), Fisher's exact test for dichotomous variables and for categories.

	**Baseline**			
**Characteristic**	**Whole cohort (n=536)**	**Eligible for baseline visit only (n=205)**	**Eligible for and completed both visits (n=331)**	**P Value (baseline only vs completed both visits)**
Age (years)	45 (11)	42 (10)	46 (11)	<0.001
Sex (n females)	389 (73%)	148 (72%)	241 (73%)	0.92
BMI (kg/m^2)	25 (23, 29)	24 (22, 27)	26 (23, 31)	<0.001
Ethnicity				0.57
White	477 (89%)	182 (89%)	295 (89%)	
Mixed	21 (4%)	9 (4%)	12 (4%)	
South Asian	24 (4%)	7 (3%)	17 (5%)	
Black	13 (2%)	7 (3%)	6 (2%)	
Healthcare worker	172 (32%)	60 (29%)	112 (34%)	0.30
At least one COVID-19 vaccination	10 (2%)	5 (2%)	5 (2%)	0.52
**Comorbidities and risks**				
Smoking				0.63
Never	349 (65%)	131 (64%)	218 (66%)	
Current	14 (3%)	7 (3%)	7 (2%)	
Ex-smoker	172 (32%)	66 (32%)	106 (32%)	
BMI				
≥25 kg/m^2	293 (55%)	93 (45%)	200 (60%)	<0.001
≥30 kg/m^2	120 (22%)	29 (14%)	91 (27%)	<0.001
Hypertension	44 (8%)	11 (5%)	33 (10%)	0.07
Diabetes	10 (2%)	3 (1%)	7 (2%)	0.75
Heart disease	9 (2%)	5 (2%)	4 (1%)	0.31
Asthma	101 (19%)	39 (19%)	62 (19%)	>0.999
Hospitalised during acute COVID-19	72 (13%)	15 (7%)	57 (17%)	0.001
Time off work (days)	56 (14, 180)	41 (13, 192)	58 (14, 150)	0.79
**15 common symptoms**				
Number reported [median (IQR)]	10 (8, 11)	10 (8, 12)	10 (8, 11)	0.50
None reported in history	0 (0%)	0 (0%)	0 (0%)	
None reported in history/ questionnaires	0 (0%)	0 (0%)	0 (0%)	
**Symptom groups**				
Systemic	245 (46%)	86 (42%)	159 (48%)	0.18
Cardiopulmonary	238 (44%)	95 (46%)	143 (43%)	0.53
Severe breathlessness (Dyspnoea 12 ≥10)	187 (36%)	67 (34%)	120 (38%)	0.40
Cognitive dysfunction	268 (50%)	108 (53%)	160 (48%)	0.37
Poor HRQoL	281 (55%)	100 (51%)	181 (57%)	0.20
None of five symptom groups	66 (13%)	29 (15%)	37 (12%)	0.34
**Duration (days: median, [IQR])**				
Initial symptoms-to-assessment	182 (132, 222)	191 (141, 230)	170 (126, 208)	0.004
COVID-19 positive-to-assessment	110 (53, 175)	115 (54, 178)	110 (56, 172)	0.422

Table S4: Characteristics for individuals with long COVID (whole cohort) at baseline, comparing those with common symptoms (systemic, cardiopulmonary, severe breathlessness, cognitive dysfunction, poor health related quality of life) and those with less common symptoms. P-value was for comparison between participants in a symptom group vs those with only less common symptoms. P-values represent results from an independent two-sample t-test (or non-parametric equivalent), Fisher's exact test for dichotomous variables and for categories.

	**Less common symptoms only**	**Systemic Symptoms**		**Cardiopulmonary Symptoms**		**Severe Breathlessness**		**Cognitive Dysfunction**		**Poor HRQoL**	
	**n=66**	**n=245**	**p-value**	**n=238**	**p-value**	**n=187**	**p-value**	**n=268**	**p-value**	**n=281**	**p-value**
**Demographic characteristics**											
Age	45 (38, 51)	44 (37, 52)	0.948	43 (38, 51)	0.557	43 (37, 50)	0.285	44 (38, 52)	0.792	43 (38, 51)	0.613
Female sex	37/66 (56%)	192/245 (78%)	**<0.001**	178/238 (75%)	**0.006**	143/187 (76%)	**0.003**	201/268 (75%)	**0.004**	224/281 (80%)	**<0.001**
BMI (kg/m^2)	25 (22, 27)	26 (23, 31)	**0.007**	26 (23, 30)	**0.015**	26 (23, 30)	**0.017**	25 (23, 30)	0.072	25 (23, 30)	0.082
**Ethnicity**											
White	53/66 (80%)	218/245 (89%)	0.095	215/238 (90%)	**0.032**	169/187 (90%)	**0.047**	242/268 (90%)	**0.032**	254/281 (90%)	**0.031**
Mixed	0/66 (0%)	3/245 (1.2%)	>0.999	3/238 (1.3%)	>0.999	4/187 (2.1%)	0.575	3/268 (1.1%)	>0.999	5/281 (1.8%)	0.588
South Asian	7/66 (11%)	10/245 (4.1%)	0.061	6/238 (2.5%)	**0.01**	2/187 (1.1%)	**0.001**	9/268 (3.4%)	**0.022**	4/281 (1.4%)	**0.001**
Black	3/66 (4.5%)	3/245 (1.2%)	0.112	3/238 (1.3%)	0.119	3/187 (1.6%)	0.185	4/268 (1.5%)	0.142	5/281 (1.8%)	0.18
Health care worker	17/66 (26%)	87/245 (36%)	0.145	71/238 (30%)	0.544	56/187 (30%)	0.636	87/268 (32%)	0.373	87/281 (31%)	0.457
**Comorbidities and risks**											
No smoker	46/66 (70%)	152/244 (62%)	0.313	143/237 (60%)	0.196	104/187 (56%)	0.058	172/268 (64%)	0.471	175/280 (62%)	0.32
Current smoker	1/66 (1.5%)	7/244 (2.9%)	>0.999	7/237 (3.0%)	>0.999	7/187 (3.7%)	0.684	8/268 (3.0%)	>0.999	8/280 (2.9%)	>0.999
Past smoker	19/66 (29%)	85/244 (35%)	0.382	87/237 (37%)	0.247	76/187 (41%)	0.104	88/268 (33%)	0.559	97/280 (35%)	0.389
BMI >25 kg/m^2	34/66 (52%)	141/245 (58%)	0.404	136/238 (57%)	0.484	104/187 (56%)	0.569	141/268 (53%)	0.891	148/281 (53%)	0.892
BMI >30 kg/m^2	7/66 (11%)	70/245 (29%)	**0.002**	59/238 (25%)	**0.012**	53/187 (28%)	**0.004**	66/268 (25%)	**0.013**	76/281 (27%)	**0.004**
Hypertension	4/66 (6.1%)	21/245 (8.6%)	0.617	21/238 (8.8%)	0.616	19/187 (10%)	0.456	24/268 (9.0%)	0.621	27/281 (9.6%)	0.475
Diabetes	1/66 (1.5%)	8/245 (3.3%)	0.69	5/238 (2.1%)	>0.999	2/187 (1.1%)	>0.999	2/268 (0.7%)	0.485	3/281 (1.1%)	0.572
Heart disease	1/66 (1.5%)	6/245 (2.4%)	>0.999	2/238 (0.8%)	0.521	5/187 (2.7%)	>0.999	3/268 (1.1%)	0.587	6/281 (2.1%)	>0.999
Asthma	8/66 (12%)	45/245 (18%)	0.272	59/238 (25%)	**0.029**	45/187 (24%)	0.052	52/268 (19%)	0.211	57/281 (20%)	0.16
Hospitalized during acute COVID-19	11/66 (17%)	41/245 (17%)	>0.999	37/238 (16%)	0.849	20/187 (11%)	0.274	31/268 (12%)	0.299	37/281 (13%)	0.434
**Common symptoms**											
Fever	33/63 (52%)	245/245 (100%)	**<0.001**	179/238 (75%)	**<0.001**	142/187 (76%)	**<0.001**	189/268 (71%)	**0.007**	210/281 (75%)	**<0.001**
Cough	43/63 (68%)	199/245 (81%)	**0.038**	194/238 (82%)	**0.036**	143/187 (76%)	0.242	212/268 (79%)	0.069	217/281 (77%)	0.146
Sore Throat	37/63 (59%)	191/245 (78%)	**0.003**	190/238 (80%)	**<0.001**	144/187 (77%)	**0.009**	201/268 (75%)	**0.013**	220/281 (78%)	**0.002**
Runny Nose	21/63 (33%)	84/245 (34%)	>0.999	97/238 (41%)	0.312	74/187 (40%)	0.453	102/268 (38%)	0.563	101/281 (36%)	0.771
Wheezing	10/63 (16%)	137/245 (56%)	**<0.001**	238/238 (100%)	**<0.001**	126/187 (67%)	**<0.001**	150/268 (56%)	**<0.001**	156/281 (56%)	**<0.001**
Chest Pain	34/63 (54%)	214/245 (87%)	**<0.001**	238/238 (100%)	**<0.001**	172/187 (92%)	**<0.001**	239/268 (89%)	**<0.001**	251/281 (89%)	**<0.001**
Myalgia	49/63 (78%)	245/245 (100%)	**<0.001**	216/238 (91%)	**0.008**	171/187 (91%)	**0.007**	242/268 (90%)	**0.01**	256/281 (91%)	**0.007**
Joint Pain	30/63 (48%)	245/245 (100%)	**<0.001**	189/238 (79%)	**<0.001**	146/187 (78%)	**<0.001**	207/268 (77%)	**<0.001**	221/281 (79%)	**<0.001**
Fatigue	59/63 (94%)	245/245 (100%)	**0.002**	237/238 (100%)	**0.007**	184/187 (98%)	0.07	267/268 (100%)	**0.005**	276/281 (98%)	0.062
Shortness of Breath	39/63 (62%)	232/245 (95%)	**<0.001**	238/238 (100%)	**<0.001**	185/187 (99%)	**<0.001**	250/268 (93%)	**<0.001**	265/281 (94%)	**<0.001**
Inability to walk	9/63 (14%)	102/245 (42%)	**<0.001**	95/238 (40%)	**<0.001**	79/187 (42%)	**<0.001**	90/268 (34%)	**0.002**	110/281 (39%)	**<0.001**
Headaches	37/63 (59%)	245/245 (100%)	**<0.001**	211/238 (89%)	**<0.001**	160/187 (86%)	**<0.001**	239/268 (89%)	**<0.001**	252/281 (90%)	**<0.001**
Seizures	0/63 (0%)	4/245 (1.6%)	0.585	1/238 (0.4%)	>0.999	2/187 (1.1%)	>0.999	3/268 (1.1%)	>0.999	4/281 (1.4%)	>0.999
Abdominal pain	21/63 (33%)	157/245 (64%)	**<0.001**	154/238 (65%)	**<0.001**	121/187 (65%)	**<0.001**	157/268 (59%)	**<0.001**	180/281 (64%)	**<0.001**
Diarrhoea	29/63 (46%)	152/245 (62%)	**0.031**	148/238 (62%)	**0.022**	112/187 (60%)	0.058	156/268 (58%)	0.091	179/281 (64%)	**0.011**
Number of common symptoms	7 (6, 9)	11 (10, 12)	**<0.001**	11 (10, 12)	**<0.001**	11 (9, 12)	**<0.001**	10 (9, 12)	**<0.001**	11 (9, 12)	**<0.001**
**MRI abnormality**											
Liver (cT1 or fat high)	13/65 (20%)	80/240 (33%)	**0.048**	69/237 (29%)	0.159	64/186 (34%)	**0.042**	77/264 (29%)	0.163	85/278 (31%)	0.096
Liver cT1 (high)	8/64 (12%)	30/238 (13%)	>0.999	25/236 (11%)	0.655	21/185 (11%)	0.822	30/263 (11%)	0.828	36/277 (13%)	>0.999
Liver fat (high)	10/66 (15%)	70/245 (29%)	**0.027**	60/238 (25%)	0.099	58/187 (31%)	**0.015**	67/267 (25%)	0.103	72/281 (26%)	0.078
Liver volume (high)	5/66 (7.6%)	19/244 (7.8%)	>0.999	16/237 (6.8%)	0.787	19/187 (10%)	0.632	18/267 (6.7%)	0.788	24/280 (8.6%)	>0.999
Pancreas (srT1 or fat high)	12/63 (19%)	51/230 (22%)	0.729	43/224 (19%)	>0.999	41/177 (23%)	0.597	47/252 (19%)	>0.999	53/264 (20%)	>0.999
Pancreas cT1 (high)	6/63 (9.5%)	25/229 (11%)	>0.999	19/223 (8.5%)	0.802	18/175 (10%)	>0.999	19/251 (7.6%)	0.605	24/263 (9.1%)	>0.999
Pancreatic fat (high)	8/66 (12%)	37/235 (16%)	0.56	34/230 (15%)	0.691	33/182 (18%)	0.334	36/259 (14%)	0.841	44/270 (16%)	0.454

	Less common symptoms only	Systemic Symptoms		Cardiopulmonary Symptoms		Severe Breathlessness		Cognitive Dysfunction		Poor HRQoL	
Kidney (cortex T1 high)	7/65 (11%)	39/241 (16%)	0.332	37/237 (16%)	0.428	34/185 (18%)	0.177	38/263 (14%)	0.548	46/278 (17%)	0.34
Kidney volume (high)	6/66 (9.1%)	26/243 (11%)	0.822	21/236 (8.9%)	>0.999	22/187 (12%)	0.653	23/266 (8.6%)	>0.999	29/279 (10%)	>0.999
Splenomegaly	3/65 (4.6%)	27/243 (11%)	0.157	23/236 (9.7%)	0.316	18/187 (9.6%)	0.298	26/267 (9.7%)	0.229	23/279 (8.2%)	0.438
Lung FAC (low)	1/56 (1.8%)	7/233 (3.0%)	>0.999	6/231 (2.6%)	>0.999	5/180 (2.8%)	>0.999	5/262 (1.9%)	>0.999	7/271 (2.6%)	>0.999
Heart injury	15/66 (23%)	42/240 (18%)	0.372	44/234 (19%)	0.486	34/183 (19%)	0.474	48/261 (18%)	0.485	56/276 (20%)	0.736
Cardiac global T1 (elevated in >=3 segments)	8/66 (12%)	17/245 (6.9%)	0.2	21/238 (8.8%)	0.477	15/187 (8.0%)	0.325	20/268 (7.5%)	0.221	22/281 (7.8%)	0.328
LF EF (low)	3/66 (4.5%)	9/243 (3.7%)	0.724	8/237 (3.4%)	0.71	7/186 (3.8%)	0.725	10/266 (3.8%)	0.727	9/280 (3.2%)	0.706
RF EF (low)	2/66 (3.0%)	8/243 (3.3%)	>0.999	11/237 (4.6%)	0.741	2/186 (1.1%)	0.281	11/266 (4.1%)	>0.999	9/280 (3.2%)	>0.999
LV EDV (high)	1/66 (1.5%)	1/243 (0.4%)	0.382	2/237 (0.8%)	0.523	1/186 (0.5%)	0.456	2/266 (0.8%)	0.487	1/280 (0.4%)	0.346
RV EDV (high)	0/66 (0%)	3/243 (1.2%)	>0.999	4/237 (1.7%)	0.58	2/186 (1.1%)	>0.999	5/266 (1.9%)	0.587	3/280 (1.1%)	>0.999
Global longitudinal strain (high)	3/66 (4.5%)	10/240 (4.2%)	>0.999	7/233 (3.0%)	0.464	8/183 (4.4%)	>0.999	9/258 (3.5%)	0.715	17/273 (6.2%)	0.775
**Multi-organ**											
no organ impaired	29/66 (44%)	95/245 (39%)	0.48	94/238 (39%)	0.571	66/187 (35%)	0.238	114/268 (43%)	0.89	113/281 (40%)	0.581
>= 1 organ impaired	37/66 (56%)	150/245 (61%)	0.48	144/238 (61%)	0.571	121/187 (65%)	0.238	154/268 (57%)	0.89	168/281 (60%)	0.581
>=2 organs impaired	12/66 (18%)	66/245 (27%)	0.154	49/238 (21%)	0.731	49/187 (26%)	0.242	56/268 (21%)	0.734	68/281 (24%)	0.333
>=3 organs impaired	2/66 (3.0%)	23/245 (9.4%)	0.125	21/238 (8.8%)	0.185	19/187 (10%)	0.116	23/268 (8.6%)	0.189	25/281 (8.9%)	0.13

.

Table S5: Blood investigations in the whole cohort and the follow-up group of individuals with long COVID. Baseline and follow-up in the follow-up group were compared using McNemar's test. Note that 'No Change' indicates that there was no change in blood value for all individuals with paired data.

	**Whole cohort**	**Follow-up group**				
**Biomarker**	**Baseline** n=536	**Baseline** n = 331	**Follow-up** n=331	**p-value (baseline vs follow-up visits)**	**Unit**	**Reference range**
Alanine transferase (high)	74/508 (15%)	49/313 (16%)	46/326 (14%)	0.391	IU/L	F: 10-35,
(low)	7/508 (1%)	4/313 (1%)	4/326 (1%)	>0.999		M: 10-50
Albumin (high)	27/508 (5%)	18/313 (1%)	11/326 (3%)	0.099	g/L	34-50
(low)	0/508 (0%)	0/313 (0%)	0/326 (0%)	No Change		
Alkaline Phosphatase (high)	13/508 (3%)	8/313 (3%)	11/326 (3%)	0.450	IU/L	F: 35-104,
(low)	13/508 (3%)	7/313 (2%)	5/326 (2%)	0.617		M: 40-129
Amylase (high)	34/463 (7%)	22/284 (8%)	22/279 (8%)	>0.999	IU/L	28 - 100
(low)	10/463 (2%)	6/284 (2%)	10/279 (4%)	0.221		
Aspartate transferase (high)	43/488 (9%)	26/296 (9%)	37/312 (12%)	0.458	IU/L	F: 0-31,
(low)	0/488 (0%)	0/296 (0%)	0/312 (0%)	No Change		M:0-37
Basophils (high)	2/508 (0%)	2/313 (1%)	7/324 (2%)	0.131	10^9/L	0.0-0.1
(low)	0/508 (0%)	0/313 (0%)	0/324 (0%)	No Change		
Bicarbonate (high)	25/508 (5%)	17/313 (5%)	21/326 (6%)	>0.999	mmol/L	22-29
(low)	49/508 (10%)	34/313 (11%)	33/326 (10%)	0.896		
Bilirubin (high)	17/508 (3%)	8/313 (3%)	12/326 (4%)	0.724	μmol/L	0-20
(low)	0/508 (0%)	0/313 (0%)	0/326 (0%)	No Change		
C-peptide (high)	19/463 (4%)	19/283 (7%)	24/279 (9%)	0.823	μg/L	1.1 - 4.4
(low)	0/463 (0%)	0/283 (0%)	1/279 (0%)	No Change		
Calcium (high)	7/508 (1%)	5/313 (2%)	1/326 (0%)	No Change	mmol/L	2.20-2.60
(low)	8/508 (2%)	4/313 (1%)	5/326 (2%)	>0.999		
Chloride (high)	10/508 (2%)	9/313 (3%)	1/326 (0%)	No Change	mmol/L	98-107
(low)	11/508 (2%)	7/313 (2%)	6/326 (2%)	0.752		
Cholesterol (high)	236/508 (46%)	152/313 (49%)	157/326 (48%)	0.791	mmol/L	<5
Creatine kinase (high)	40/508 (8%)	26/313 (8%)	41/323 (13%)	0.059	IU/L	F: 26-104,
(low)	2/508 (0%)	1/313 (0%)	2/323 (1%)	>0.999		M: 38-204
Creatinine (high)	6/508 (1%)	5/313 (2%)	6/326 (2%)	>0.999	μmol/L	F: 49-92,
(low)	25/508 (5%)	14/313 (4%)	16/326 (5%)	0.628		M: 66-112
CRP - high sensitivity (high)	37/507 (7%)	29/312 (2%)	30/326 (9%)	>0.999	mg/L	<5
eGFR (low)	5/508 (1%)	4/313 (1%)	6/325 (2%)	>0.999	mL/min/1.73m2	> 60
Eosinophils (high)	14/508 (3%)	7/313 (2%)	11/324 (3%)	0.546	10^9/L	0-0.4
(low)	0/508 (0%)	0/313 (0%)	0/324 (0%)	No Change		
ESR (high)	40/510 (8%)	33/313 (11%)	28/323 (9%)	0.286	mm/hr	M: 1-20,
(low)	0/510 (0%)	0/313 (0%)	0/323 (0%)	No Change		F or >40 years: 1-23
Gamma GT (high)	32/508 (6%)	21/313 (7%)	18/326 (6%)	0.267	IU/L	F: 6-42,
(low)	12/508 (2%)	5/313 (2%)	4/326 (1%)	>0.999		M: 10-71
Globulin (high)	2/508 (0%)	1/313 (0%)	0/326 (0%)	No Change	g/L	19-35
(low)	14/508 (3%)	9/313 (3%)	11/326 (3%)	0.814		
Haemoglobin (high)	6/508 (1%)	5/313 (2%)	7/324 (2%)	>0.999	g/L	F: 115-115,
(low)	13/508 (3%)	7/313 (2%)	6/324 (2%)	>0.999		M: 130-170
HCT (high)	10/508 (2%)	9/313 (3%)	12/324 (4%)	0.752		F: 0.33-0.45,
(low)	8/508 (2%)	4/313 (1%)	1/324 (0%)	0.248		M: 0.37-0.5
HDL Cholesterol (high)	176/508 (35%)	104/313 (33%)	101/326 (31%)	0.371	mmol/L	F: 1.2-1.7,
(low)	40/508 (8%)	31/313 (10%)	31/326 (10%)	>0.999		M: 0.9-1.5
Insulin (high)	41/460 (9%)	30/281 (11%)	27/276 (10%)	0.646	mIU/L	2.6-24.9
(low)	10/460 (2%)	2/281 (1%)	7/276 (3%)	**0.041**		
Iron (high)	24/508 (5%)	16/313 (5%)	11/326 (3%)	0.404	μmol/L	F: 6.6-26,
(low)	10/508 (2%)	5/313 (2%)	11/326 (3%)	0.267		M: 10.6-28.3
LDH (high)	80/500 (16%)	59/306 (19%)	70/319 (22%)	0.688	IU/L	F: 135-214,
(low)	19/500 (4%)	9/306 (3%)	10/319 (3%)	>0.999		M: 38-204
LDL Cholesterol (high)	167/500 (33%)	108/306 (35%)	116/325 (36%)	>0.999	mmol/L	<3
Lymphocytes (high)	2/508 (0%)	2/313 (1%)	6/324 (2%)	0.371	10^9/L	1.2-3.65
(low)	38/508 (7%)	25/313 (8%)	28/324 (9%)	0.689		
Magnesium (high)	2/508 (0%)	2/313 (1%)	4/326 (1%)	>0.999	mmol/L	0.6-1.0
(low)	1/508 (0%)	1/313 (0%)	1/326 (0%)	No Change		
MCH (high)	4/508 (1%)	3/313 (1%)	5/324 (2%)	>0.999	pg	26-33.5
(low)	8/508 (2%)	6/313 (2%)	8/324 (2%)	>0.999		
MCHC (high)	106/508 (21%)	62/313 (20%)	49/324 (15%)	**0.050**	g/L	300-350
(low)	0/508 (0%)	0/313 (0%)	1/324 (0%)	No Change		
MCV (high)	1/508 (0%)	1/313 (0%)	3/324 (1%)	0.480	fL	80-99
(low)	10/508 (2%)	8/313 (3%)	6/324 (2%)	0.683		
Monocytes (high)	4/508 (1%)	3/313 (1%)	2/324 (1%)	>0.999	10^9/L	0.2-1
(low)	2/508 (0%)	0/313 (0%)	0/324 (0%)	No Change		
MPV (high)	8/506 (2%)	6/313 (2%)	7/324 (2%)	0.617	fL	7-13
(low)	0/506 (0%)	0/313 (0%)	0/324 (0%)	No Change		
Neutrophils (high)	8/508 (2%)	7/313 (2%)	7/324 (2%)	>0.999	10^9/L	2-7.5
(low)	30/508 (6%)	13/313 (4%)	17/324 (5%)	0.814		
Phosphate (high)	13/508 (3%)	6/313 (2%)	6/326 (2%)	>0.999	mmol/L	0.87-1.45
(low)	53/508 (10%)	38/313 (12%)	34/326 (10%)	0.542		
Platelet count (high)	22/505 (4%)	16/311 (5%)	15/324 (5%)	>0.999	10^9/L	150-400
(low)	2/505 (0%)	1/311 (0%)	2/324 (1%)	>0.999		
Potassium (high)	231/476 (49%)	140/289 (48%)	91/248 (37%)	**0.016**	mmol/L	3.5-5.1
(low)	0/476 (0%)	0/289 (0%)	0/248 (0%)	No Change		

	Whole cohort	Follow-up group				
**Biomarker**	**Baseline** n=536	**Baseline** n = 331	**Follow-up** n=331	**p-value (baseline vs follow-up visits)**	**Unit**	**Reference range**
RDW (high)<br>(low)	12/507 (2%)<br>26/507 (5%)	10/312 (3%)<br>17/312 (5%)	5/324 (2%)<br>8/324 (2%)	**0.041**<br>**0.027**		11.5-15.0
Red cell count (high)<br>(low)	14/508 (3%)<br>17/508 (3%)	9/313 (3%)<br>8/313 (3%)	6/324 (2%)<br>12/324 (4%)	0.450<br>0.546	10^12/L	F: 3.95-5.15,<br>M: 4.4-5.8
Sodium (high)<br>(low)	1/508 (0%)<br>17/508 (3%)	1/313 (0%)<br>10/313 (3%)	2/326 (1%)<br>8/326 (2%)	>0.999<br>0.579	mmol/L	135-145
Testosterone (high)<br>(low)	19/463 (4%)<br>9/463 (2%)	10/284 (4%)<br>5/284 (2%)	7/279 (3%)<br>7/279 (3%)	0.579<br>0.724	nmol/L	F: 0-1.8,<br>M: 7.6 - 31.4
Thyroid stimulating hormone (high)<br>(low)	3/468 (1%)<br>0/468 (0%)	3/288 (1%)<br>0/288 (0%)	4/279 (1%)<br>1/279 (0%)	>0.999<br>No Change	mIU/L	0.27-4.2
TIBC (high)<br>(low)	19/501 (4%)<br>1/501 (0%)	8/307 (3%)<br>1/307 (0%)	5/319 (2%)<br>5/319 (2%)	0.221<br>0.134	μmol/L	41-77
Total protein (high)<br>(low)	2/508 (0%)<br>7/508 (1%)	1/313 (0%)<br>6/313 (2%)	0/326 (0%)<br>9/326 (3%)	No Change<br>0.773	g/L	63-83
Transferrin saturation (high)<br>(low)	9/501 (2%)<br>79/501 (16%)	6/307 (2%)<br>55/307 (18%)	6/319 (2%)<br>65/319 (20%)	>0.999<br>0.222	%	20-55
Triglycerides (high)	71/508 (14%)	49/313 (16%)	44/326 (13%)	0.349	mmol/L	<2.3
Troponin I (high)	4/463 (1%)	3/184 (1%)	2/279 (1%)	>0.999	ng/L	< 15.6
Urea (high)<br>(low)	1/508 (0%)<br>1/508 (0%)	0/313 (0%)<br>0/313 (0%)	3/326 (1%)<br>1/326 (0%)	No Change<br>No Change	mmol/L	1.7-8.3
Uric acid (high)<br>(low)	29/508 (6%)<br>59/508 (12%)	22/313 (7%)<br>31/313 (10%)	19/326 (6%)<br>27/326 (8%)	0.453<br>0.571	μmol/L	F: 175-363,<br>M: 266-474
White cell count (high)<br>(low)	19/508 (4%)<br>1/508 (0%)	14/313 (4%)<br>0/313 (0%)	16/324 (5%)<br>0/324 (0%)	0.814<br>No Change	10^9/L	3-10

Table S6: Comparison of MRI metrics between healthy controls and whole long COVID cohort at baseline. Comparisons are statistically assessed using the two-sample t-test for normal data and the two-sample Wilcox test for non-normal data. Normality is assessed using the Shapiro-Wilk test.

Metric	N total	Healthy controls (n=92)	Long COVID (n=536)	P-value
**CARDIAC**				
(Field strength independent metrics)				
**Global longitudinal strain 3D (%)**	601	-14.68 (2.4)	-14.50 (2.3)	0.336
Missing		13	14	
**RV EF (%)**	621	57.6 (4.5)	59.1 (4.9)	**0.004**
Missing		5	2	
**LV EF (%)**	625	59.5 (56.6, 62.7)	59.7 (56.8, 62.4)	0.747
Missing		1	2	
**RV EDV (ml)** (BSA corrected)	621	87 (78 101)	77 (68 87)	**<0.001**
Missing		5	2	
**LV EDV (ml)** (BSA corrected)	593	86 (78, 99)	80 (70, 90)	**<0.001**
Missing		5	2	
(Field strength dependent: 1.5T)				
**Global T1 (ms)**	387	968 (962, 988)	976 (956, 991)	0.592
Missing		1	9	
(Field strength dependent: 3T)				
**Global T1 (ms)**	224	1,179 (1,152, 1,199)	1,182 (1,159, 1,199)	0.972
Missing		0	7	
**LIVER**				
(Field strength independent metrics)				
**cT1 (ms)**	613	709 (667, 748)	714 (669, 759)	0.304
Missing		3	12	
**PDFF (%)**	578	1.8 (1.3, 2.6)	2.6 (1.6, 5.0)	**<0.001**
Missing		3	1	
**Liver volume (ml)**	626	1,344 (1,238, 1,550)	1,420 (1,269, 1,636)	0.126
Missing		1	1	
**KIDNEY**				
(Field strength independent metrics)				
**Left volume (ml)**	624	141 (125, 170)	149 (129, 169)	0.430
Missing		2	2	
**Right volume (ml)**	622	151 (131, 178)	149 (132, 168)	0.392
Missing		2	4	
(Field strength dependent: 1.5T)				
**Left cortex T1 (ms)**	394	1,065 (54)	1,080 (71)	0.070
Missing		1	2	
**Right cortex T1 (ms)**	395	1,050 (58)	1,070 (68)	**0.020**
Missing		1	1	
(Field strength dependent: 3T)				
**Left cortex T1 (ms)**	227	1,397 (61)	1,412 (75)	0.213
Missing		0	4	
**Right cortex T1 (ms)**	225	1,389 (69)	1,389 (79)	0.969
Missing		0	6	
**PANCREAS**				
(Field strength independent metrics)				
**srT1 (ms)**	590	714 (686, 743)	717 (683, 761)	0.390
Missing		6	32	
**PDFF (%)**	607	2.11 (1.62, 2.91)	2.80 (2.10, 4.75)	**<0.001**
Missing		4	17	
**SPLEEN**				
(Field strength independent metrics)				
**Volume (ml)**	624	182 (121, 239)	182 (146, 240)	0.248
Missing		1	3	
**LUNG**				
(Field strength independent metrics)				
**Deep fractional area change (%)**	582	47 (39, 53)	45 (38, 51)	0.120
Missing		17	29	

Table S7: Associations between biomarkers and symptom groups in individuals with long COVID. Odds ratios (95% CI) and p-values from stepwise-selected model (using AIC) are reported. The timepoint 'prediction' refers to an association of biomarker at baseline and symptom at follow-up. Sample sizes for presenting the outcome (yes/no) refer to observations with no missing data. In bold are significant biomarkers identified in the combined regression model.

**Symptom group**	**Timepoint**	**MRI metrics (stepwise model)**	**Blood metrics (stepwise model)**	**Combined (stepwise model)**
**Severe breathlessness**	Baseline (whole cohort)	Liver fat: OR 1.4 (1.13, 1.73), p=0.002<br>Pancreatic fat: OR 1.22 (0.98, 1.51), p=0.07<br>Kidney cortex T1: OR 1.17 (0.95, 1.44), p=0.128<br>Liver cT1: OR 0.82 (0.65, 1.03), p=0.095<br>Age: OR: 0.69 (0.56, 0.86), p=0.001<br>Sex (male): OR 0.59 (0.36, 0.94), p=0.029<br>Sample size: yes n=161, no, n=278	Transferrin sat (low): OR 1.32 (1.08, 1.62), p=0.007<br>Cholesterol (high): OR 1.25 (1, 1.56), p=0.046<br>MCHC (high): OR 1.2 (0.98, 1.47), p=0.082<br>Age: OR 0.79 (0.63, 0.98), p=0.037<br>Sample size: yes n=150, no, n=248	**Liver fat: OR 1.40 (1.12, 1.75), p=0.003**<br>Transferrin sat (low): OR 1.24 (0.99, 1.55), p=0.057<br>MCHC (high): OR 1.2 (0.95, 1.51) p=0.132<br>Age: OR 0.8 (0.63, 1) p=0.055<br>Sex (male): OR 0.64 (0.36, 1.12) p=0.127<br>Sample size: yes n=133, no, n=214
	follow-up (follow-up group)	Liver volume: OR 1.42 (1.02, 2.00), p=0.036<br>Age: OR 0.69 (0.49, 0.95), p=0.025<br>Kidney volume: OR 0.68 (0.40, 1.00), p=0.083<br>Sample size: yes n=59, no, n=129	BMI: OR 1.39 (1.03, 1.91) p=0.036<br>Potassium (high): OR 1.3 (0.95, 1.78) p=0.099<br>Age: OR 0.78 (0.57, 1.07) p=0.128<br>Transferrin sat (low): OR 0.72 (0.51, 1) p=0.061<br>Sex (male): OR 0.45 (0.2, 0.96) p=0.044<br>Sample size: yes n=64, no, n=128	**BMI: OR 1.73 (1.11, 2.77) p=0.018**<br>Age: OR 0.69 (0.46, 1.02) p=0.067<br>**Transferrin sat (low): OR 0.60 (0.37, 0.92) p=0.027**<br>Sex (male): OR 0.46 (0.18, 1.14) p=0.101<br>**Kidney Volume: OR 0.45 (0.18, 0.79) p=0.019**<br>Sample size: yes n=44, no, n=85
	prediction (follow-up group)	Liver fat: OR 1.40 (1.06, 1.85), p=0.016<br>Lungs FAC: OR 1.23 (0.95, 1.67), p=0.124<br>Age: OR 0.72 (0.53, 0.96), p=0.026<br>Sex (male): OR 0.48 (0.24, 0.92), p=0.033<br>Sample size: yes n=79, no, n=178	Cholesterol (high): OR 1.51 (1.02, 2.25), p=0.042<br>BMI: OR 1.3 (0.96, 1.76), p=0.087<br>Age: OR 0.77 (0.56, 1.05), p=0.108<br>LDH (high): OR 0.76 (0.53, 1.04), p=0.106<br>LDL Cholesterol (high): OR 0.75 (0.5, 1.1), p=0.142<br>Sample size: yes n=71, no, n=156	**Liver Fat: OR 1.55 (1.1, 2.2) p=0.013**<br>Cholesterol (high): OR 1.33 (0.93, 1.92), p=0.124<br>MCHC (high): OR 1.3 (0.92, 1.84) p=0.128<br>**Age: OR 0.64 (0.43, 0.93) p=0.021**<br>**Liver volume: OR 0.62 (0.39, 0.9) p=0.021**<br>**Sex (male): OR 0.26 (0.09, 0.66) p=0.007**<br>Sample size: yes n=60, no, n=134
**Cognitive dysfunction**	Baseline (whole cohort)	BMI: OR 1.15 (0.95, 1.39) p=0.148<br>Pancreas srT1: OR 0.84 (0.69, 1.01) p=0.066<br>Sample size: yes n=236, no, n=222	Potassium (high): OR 1.36 (1.12, 1.66), p=0.002<br>Sample size: yes n=218, no, n=195	Potassium (high): OR 1.44 (1.16, 1.78) p=0.001<br>Spleen Volume: OR 1.23 (0.98, 1.55) p=0.078<br>Pancreas srT1: OR 0.85 (0.68, 1.05) p=0.127<br>Sample size: yes n=192, no, n=167
	follow-up (follow-up group)	Liver cT1: OR 1.48 (1.09, 2.05) p=0.015<br>Kidney cortex T1: OR 0.75 (0.53, 1.02) p=0.078<br>Pancreatic fat: OR 0.62 (0.42, 0.87), p=0.009<br>Sex (male): OR 0.37 (0.17, 0.76) p=0.009<br>Sample size: yes n=79, no n=123	CK (high): OR 0.79 (0.57, 1.07) p=0.14<br>Sex (male): OR 0.51 (0.26, 0.99) p=0.05<br>Sample size: yes n=83, no, n=123	MCHC (high): OR 1.38 (0.94, 2.08) p=0.103<br>Liver cT1: OR 1.37 (0.92, 2.07) p=0.122<br>Cardiac T1: OR 0.75 (0.5, 1.1) p=0.15<br>Pancreas Fat: OR 0.67 (0.41, 1.01) p=0.073<br>CK (high): OR 0.66 (0.4, 1) p=0.067<br>**Kidney cortex T1: OR 0.66 (0.43, 0.96) p=0.037**<br>**Sex (male): OR 0.29 (0.1, 0.72) p=0.011**<br>Sample size: yes n=56, no, n=82
	Prediction	BMI: OR 0.8 (0.62, 1.02) p=0.084	BMI: 0.75 (0.56, 0.97) p=0.036	**MCHC (high): OR 1.43 (1.04, 2) p=0.032**

Symptom group	Timepoint	MRI metrics (stepwise model)	Blood metrics (stepwise model)	Combined (stepwise model)
	(follow-up group)	Sex (male): OR 0.41 (0.22, 0.74) p=0.004<br>Sample size: yes n=104, no n=175	Sex (male): OR 0.39 (0.19, 0.73) p=0.005<br>Sample size: yes n=101, no n=146	Age: OR 1.35 (0.98, 1.88) p=0.07<br>Cardiac T1: OR 1.29 (0.95, 1.78) p=0.107<br>Cholesterol (high): OR 0.79 (0.58, 1.09) p=0.152<br>Pancreas srT1: OR 0.79 (0.56, 1.08) p=0.155<br>**BMI: OR 0.68 (0.49, 0.93) p=0.019**<br>**Sex (male): OR 0.21 (0.08, 0.48) p<0.001**<br>Sample size: yes n=56, no, n=82
**Poor HRQoL**	Baseline (whole cohort)	BMI: OR 1.21 (0.99, 1.49) p=0.073<br>Cardiac T1: OR 1.16 (0.95, 1.42) p=0.143<br>Age: OR 0.84 (0.69, 1.02) p=0.074<br>Sex (male): OR 0.43 (0.28, 0.67) p<0.001<br>Sample size: yes n=241, no n=202	Sex (male): OR 0.44 (0.27, 0.7) p=0.001<br>Sample size: yes n=231, no n=167	Liver Fat: OR 1.21 (0.97, 1.52) p=0.103<br>**Sex (male): OR 0.42 (0.25, 0.7) p=0.001**<br>Sample size: yes n=200, no, n=147
	follow-up (follow-up group)	Liver Volume: OR 1.74 (1.25, 2.59) p=0.002<br>Age: OR 0.75 (0.55, 1.02) p=0.069<br>Cardiac T1: OR 0.71 (0.5, 0.98) p=0.043<br>Sample size: yes n=84, no n=104	MCHC (high): OR 1.28 (0.94, 1.78) p=0.122<br>Transferrin sat (low): OR 0.73 (0.53, 0.99) p=0.048<br>Cholesterol (high): OR 0.7 (0.51, 0.95) p=0.023<br>Age: OR 0.67 (0.49, 0.92) p=0.014<br>Sex (male): OR 0.35 (0.16, 0.92) p=0.014<br>Sample size: yes n=93, no n=99	**Liver volume: OR 1.77 (1.15, 2.93) p=0.015**<br>**MCHC (high): OR 1.66 (1.09, 2.66) p=0.024**<br>**Potassium (high): OR 1.59 (1.07, 2.41) p=0.023**<br>Age: OR 0.73 (0.49, 1.08) p=0.119<br>Cardiac T1: OR 0.68 (0.42, 1.04) p=0.087<br>**Transferrin sat (low): OR 0.55 (0.34, 0.84) p=0.009**<br>**Sex (male): OR 0.32 (0.12, 0.83) p=0.023**<br>Sample size: yes n=60, no n=69
	Prediction (follow-up group)	Kidney cortex T1: OR 1.28 (0.98, 1.67) p=0.067<br>Liver cT1: OR 1.22 (0.94, 1.59) p=0.142<br>Age: OR 0.74 (0.57, 0.96) p=0.025<br>Sex (male): OR 0.53 (0.29, 0.96) p=0.039<br>Sample size: yes n=115, no n=142	C-Peptide (high): OR 1.27 (0.97, 1.72) p=0.099<br>Sex (male): OR 0.53 (0.28, 1.01) p=0.056<br>Sample size: yes n=111, no n=116	Kidney cortex T1: OR 1.28 (0.96, 1.74) p=0.1<br>Sex (male): 0.43 (0.2, 0.88) p=0.024<br>Sample size: yes n=92, no n=102

Figure S1: Example MRI data segmentations used in organ morphology measurements.

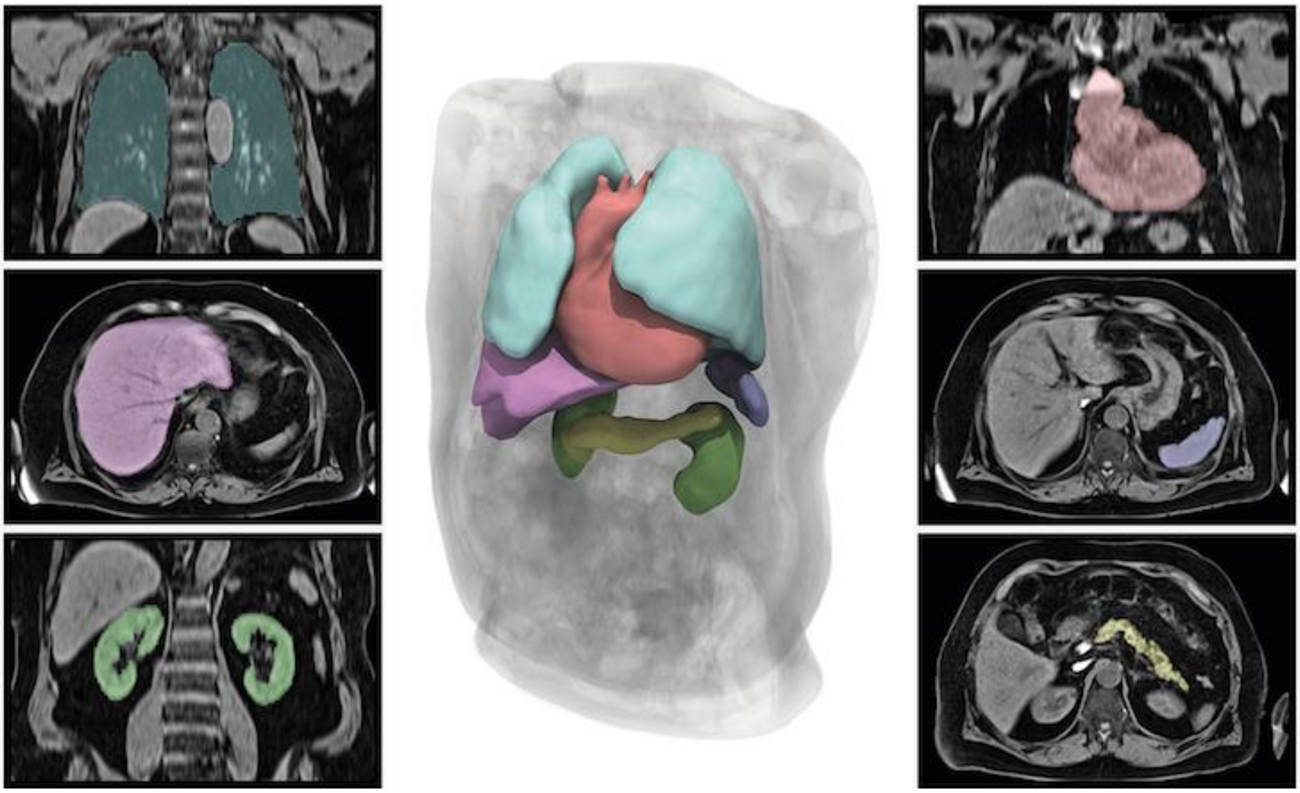

Figure S2: Symptoms at baseline in long COVID individuals with organ impairment identified at baseline, eligible for follow-up (the follow-up group) vs those without impairment, who were only invited for the baseline visit.

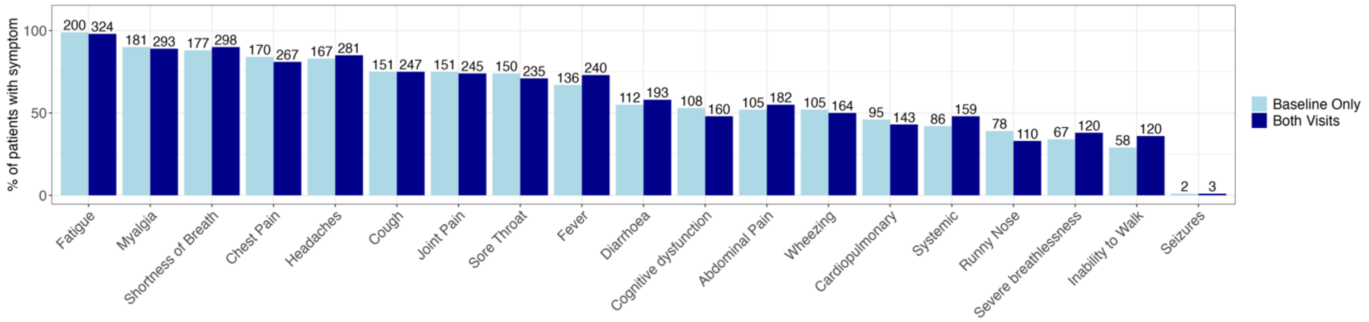

Figure S3: Self-reported health related quality of life as reported from the EQ-5D-5L instrument showing the UK specific index scores (left) and the visual analogue score (right), in the follow-up group. Comparisons were performed using the paired Wilcoxon test.

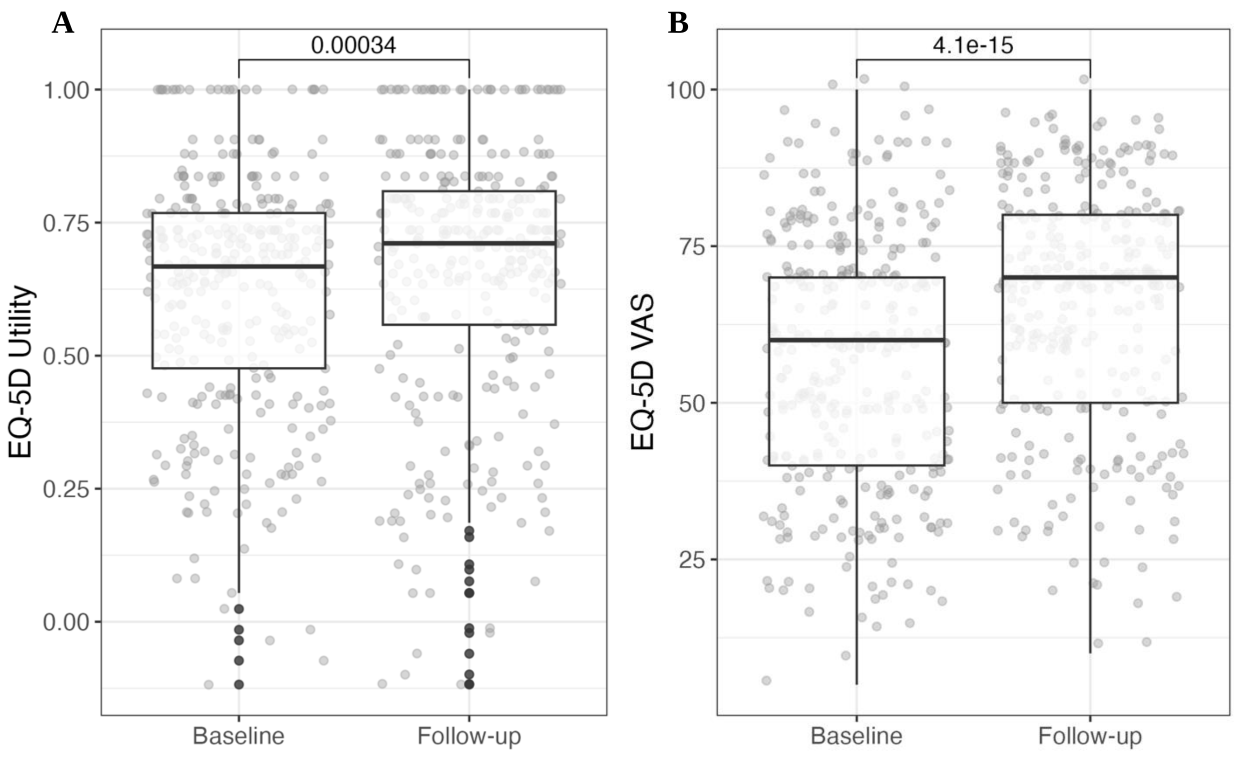

Figure S4: Dimensions of health from the EQ-5D-5L questionnaire in the follow-up group. Comparisons between baseline and follow-up were performed using McNemar's test.

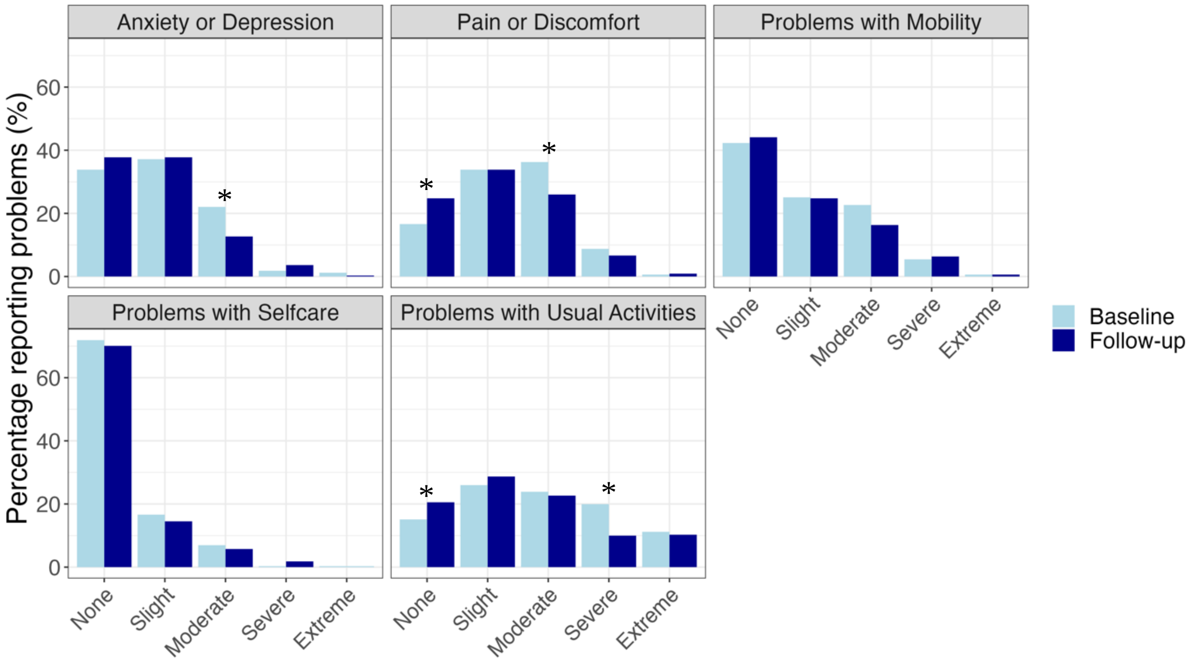

Figure S5: Proportion of long COVID cases with liver steatosis by symptom group (systemic, cardiopulmonary, severe breathlessness, brain fog, poor HRQoL. A: Whole cohort at baseline (n=536) B: A subgroup of cases from the whole cohort at baseline without metabolic syndrome (BMI>=30 or diabetes or hypertension) (n=144).

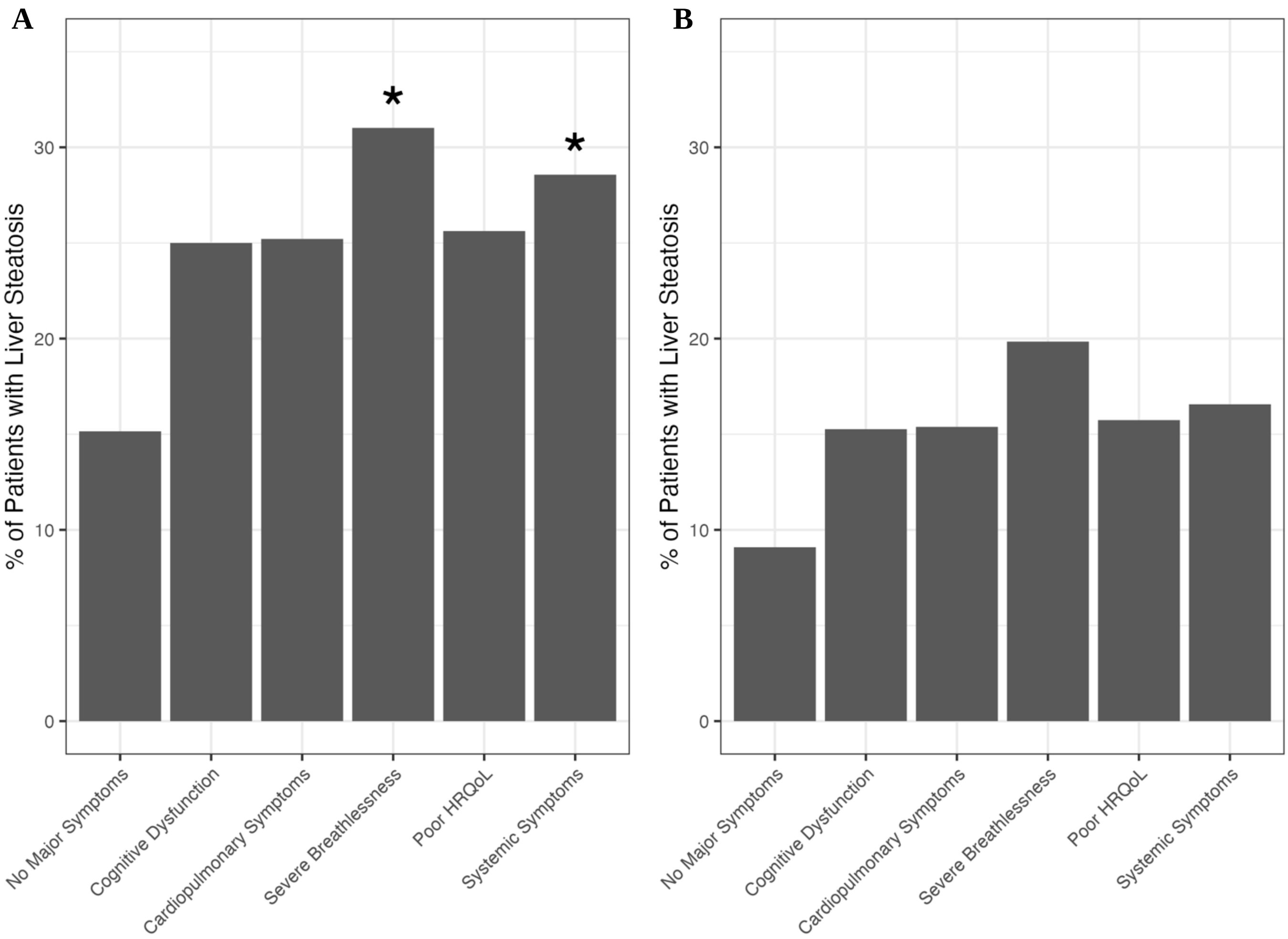

Figure S6: Heat maps showing the proportion of those with impairment in individual organs that reported specific symptoms at baseline (left) and follow-up (right) in the follow-up group. Rows and columns are sorted by mean prevalence of the symptom at baseline. Labels of the x-axis indicate the number of patients presenting with organ impairment at each time point. Shortness of breath is self-reported, and breathlessness is based on Dyspnoea-12. Greener colours indicate a higher proportion as defined by the scale on the right.

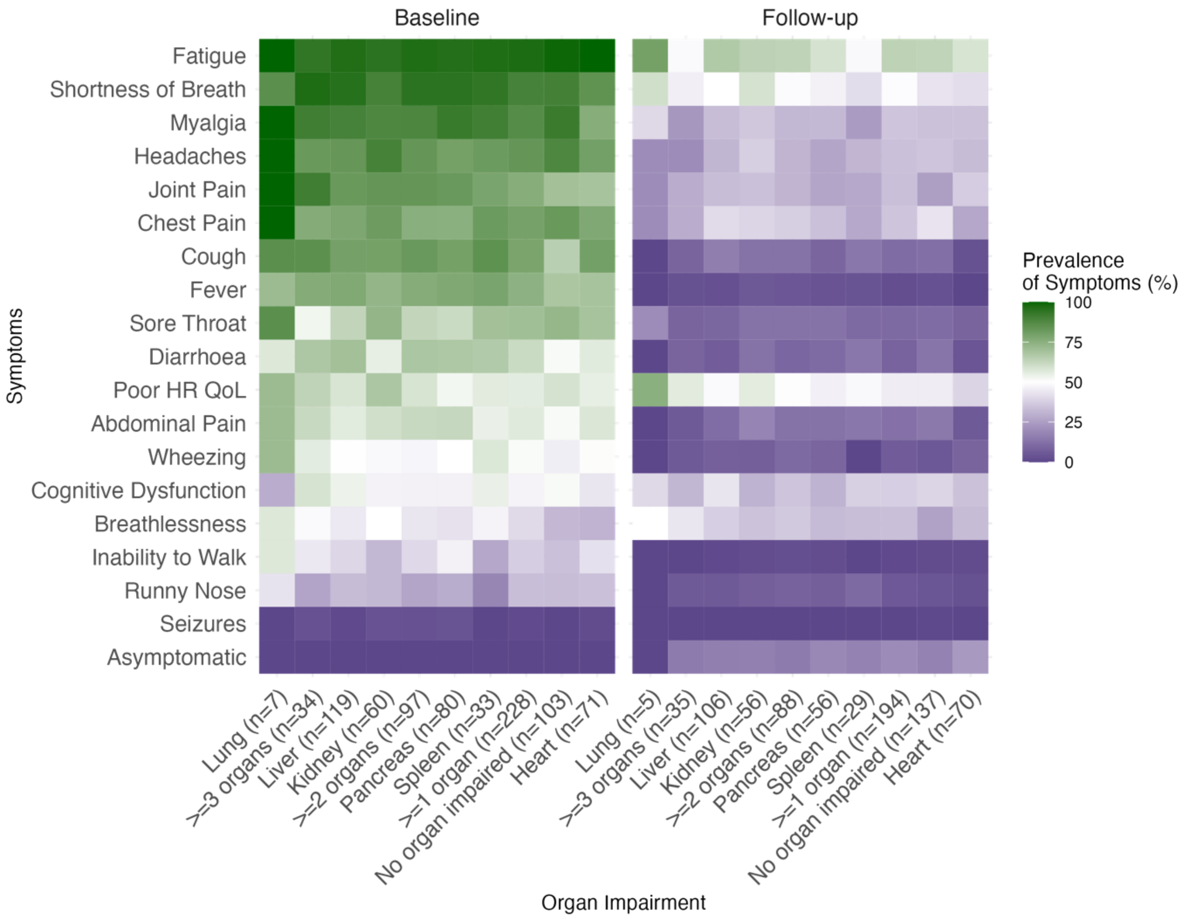

Supplementary References

1 Petersen SE, Matthews PM, Francis JM, *et al.* UK Biobank's cardiovascular magnetic resonance protocol. *J Cardiovasc Magn Reson* 2016;**18**:8. doi:10.1186/s12968-016-0227-4

2 Pavlides M, Banerjee R, Tunnicliffe EM, *et al.* Multiparametric magnetic resonance imaging for the assessment of non-alcoholic fatty liver disease severity. *Liver Int* 2017;**37**:1065–73. doi:10.1111/liv.13284

3 Petersen SE, Aung N, Sanghvi MM, *et al.* Reference ranges for cardiac structure and function using cardiovascular magnetic resonance (CMR) in Caucasians from the UK Biobank population cohort. *J Cardiovasc Magn Reson* 2017;**19**:18. doi:10.1186/s12968-017-0327-9

4 Cerqueira MD, Weissman NJ, Dilsizian V, *et al.* Standardized myocardial segmentation and nomenclature for tomographic imaging of the heart. A statement for healthcare professionals from the Cardiac Imaging Committee of the Council on Clinical Cardiology of the American Heart Association. *Circulation* 2002;**105**:539–42. doi:10.1161/hc0402.102975

5 Triay Bagur A, Aljabar P, Ridgway GR, *et al.* Pancreas MRI Segmentation Into Head, Body, and Tail Enables Regional Quantitative Analysis of Heterogeneous Disease. *Journal of Magnetic Resonance Imaging*;**n/a**. doi:10.1002/jmri.28098

6 Sudlow C, Gallacher J, Allen N, *et al.* UK biobank: an open access resource for identifying the causes of a wide range of complex diseases of middle and old age. *PLoS medicine* 2015;**12**:e1001779–e1001779. doi:10.1371/journal.pmed.1001779

7 Glodny B, Unterholzner V, Taferner B, *et al.* Normal kidney size and its influencing factors - a 64-slice MDCT study of 1.040 asymptomatic patients. *BMC Urology* 2009;**9**:19–19. doi:10.1186/1471-2490-9-19

8 Andersson A, Kelly M, Imajo K, *et al.* Clinical utility of MRI biomarkers for identifying NASH patients' high risk of progression: A multi-center pooled data and meta-analysis. *Clin Gastroenterol Hepatol* 2021;:S1542-3565(21)01056-9. doi:10.1016/j.cgh.2021.09.041

9 Bachtiar V, Kelly MD, Wilman HR, *et al.* Repeatability and reproducibility of multiparametric magnetic resonance imaging of the liver. *PLoS One* 2019;**14**. doi:10.1371/journal.pone.0214921
